# Predicting changes to *I*_Na_ from missense mutations in human *SCN5A*

**DOI:** 10.1038/s41598-018-30577-5

**Published:** 2018-08-24

**Authors:** Michael Clerx, Jordi Heijman, Pieter Collins, Paul G. A. Volders

**Affiliations:** 1 Department of Cardiology, Cardiovascular Research Institute Maastricht, Maastricht University Medical Center, Maastricht, 6202 AZ The Netherlands; 2 BioInformatics and BioMathematics, Department of Data Science and Knowledge Engineering, Maastricht University, Maastricht, 6200 MD The Netherlands

## Abstract

Mutations in *SCN5A* can alter the cardiac sodium current *I*_Na_ and increase the risk of potentially lethal conditions such as Brugada and long-QT syndromes. The relation between mutations and their clinical phenotypes is complex, and systems to predict clinical severity of unclassified *SCN5A* variants perform poorly. We investigated if instead we could predict changes to *I*_Na_, leaving the link from *I*_Na_ to clinical phenotype for mechanistic simulation studies. An exhaustive list of nonsynonymous missense mutations and resulting changes to *I*_Na_ was compiled. We then applied machine-learning methods to this dataset, and found that changes to *I*_Na_ could be predicted with higher sensitivity and specificity than most existing predictors of clinical significance. The substituted residues’ location on the protein correlated with channel function and strongly contributed to predictions, while conservedness and physico-chemical properties did not. However, predictions were not sufficiently accurate to form a basis for mechanistic studies. These results show that changes to *I*_Na_, the mechanism through which *SCN5A* mutations create cardiac risk, are already difficult to predict using purely *in-silico* methods. This partly explains the limited success of systems to predict clinical significance of *SCN5A* variants, and underscores the need for functional studies of *I*_Na_ in risk assessment.

## Introduction

The human gene *SCN5A* encodes the pore-forming *α*-subunit of the cardiac sodium channel Na_v_1.5, which carries *I*_Na_, the fast sodium current. *I*_Na_ is responsible for the initial rapid upstroke of the cellular action potential (AP) and a major determinant of electrical propagation in the heart^1^. Mutations in *SCN5A* can alter both the magnitude and the shape of *I*_Na_, which – in conjunction with other factors – can predispose carriers to clinical phenotypes including Brugada syndrome, long-QT syndrome, and conduction disorders^2^. These conditions increase the risk of potentially lethal cardiac arrhythmias and have been associated with hundreds of *SCN5A* mutations^3–6^.

New unclassified variants are regularly found, but predicting their pathogenicity has proven an exceedingly difficult task. For example, Leong *et al*.^7^ assessed the performance of commonly used *in-silico* prediction tools (PolyPhen-2, SNPs&GO, SIFT, PROVEAN, and SNAP), on variants in *SCN5A* and the potassium channel genes *KCNQ1* and *KCNH2*. They found that *KCNQ1* and *KCNH2* predictions were generally good, while predictions of *SCN5A* pathogenicity were little better than chance – as evinced by low values for the area under the receiver-operating curve (AUC) and the Matthews correlation coefficient (MCC). Similarly, Brunklaus *et al*.^8^ reviewed variants and phenotypes in all human voltage-gated sodium channel types. For *SCN5A* variants they found that, while there were some correlations, the “exact biophysical impact” of variants could not be predicted, and clinical patient management should instead be based on functional *in-vitro* or *in-vivo* studies.

One reason for the poor *SCN5A* performance of these statistical *machine-learning*^9^ methods may be that they make no use of mechanistic knowledge of cardiac electrophysiology. Instead, they take information about variants, for example the site of the substitution, the prevalence of this variant in the population, or the physico-chemical properties of the affected amino acids, and then try to establish a direct, ‘black-box’ link to pathogenicity. In several cases, however, the proarrhythmic *mechanisms* associated with a specific *SCN5A* mutation have been established using a systems approach that follows genetic screening by current measurements in expression systems, and then uses mathematical modelling to show how they can lead to the observed cell, tissue, and ECG effects^10–12^. While this approach has traditionally been applied to investigate single mutations with known clinical phenotypes, a study on long-QT syndrome type 1 by Hoefen *et al*.^13^ clearly demonstrated that (1) this approach can be extended to cover multiple mutations (provided their effects on the ion current are known), and (2) that the resulting models can predict clinical outcomes and improve risk stratification. Moreover, mechanistic models make it possible to evaluate the arrhythmogenic potential of *SCN5A* variants under different conditions, since it is well-accepted that, e.g., heart rate and autonomic tone play a critical modulating role^14^. Similarly, it is increasingly clear that the diseases associated with *SCN5A* are not monogenic, and may involve a large number of modifier genes^15^; mechanistic models can be made to include these effects, creating models personalised to a specific genetic context. It seems pertinent then to incorporate such mechanistic modelling into *in-silico* pathogenicity predictions.

In the present study, we aimed to establish whether machine-learning methods could be used to predict functional changes to *I*_Na_ from non-synonymous missense mutations in *SCN5A*. If successful, this reduced machine-learning step (from gene to ion current) could then be followed by a mechanistic modelling step (from ion current to clinical phenotype) to create a two-stage *in-silico* predictor. In addition to new capabilities (e.g., incorporating patient-specific co-morbidities) this approach would move factors such as non-linear ion-current interactions and action-potential propagation from the first, machine-learning stage, to the secondary mechanistic modelling stage, where they could be addressed more naturally. For the first stage, which is the one we address in this study, we expected the resulting reduction in complexity to lead to a significant improvement in the quality of predictions.

To see if we could predict changes to *I*_Na_ resulting from mutations, we conducted a comprehensive literature review of reported variants in *SCN5A* for which cellular electrophysiology (EP) data was available. For each variant, we collected a set of *features*, such as the substitution site (including aspects such as proximity to the voltage sensor), physico-chemical properties of the substituted amino acids (e.g., differences in charge, differences in hydrophobicity, and their Grantham similarity score, see “Machine-learning features”), and the conservedness of that site between human channel isoforms.

**Figure 1:**
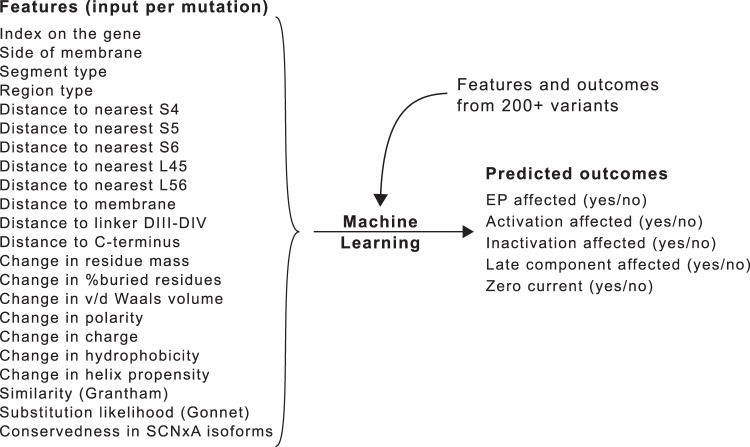
Study design: predicting changes to *I*_Na_ (outcomes) from selected features of *SCN5A* amino-acid substitutions. Different types of information were used, including data from the substitution site, physico-chemical properties of the substituted amino acids, and conservedness of the original residue in similar channels. Distances were not measured spatially, but by counting the number of residues between any two points on the protein. Prevalence of variants in the population was not explicitly included, although we did incorporate a population-based mutation probability (Gonnet score). Each outcome was assessed independently, resulting in a system of five independent binary classifiers.

As *outcomes*, we noted when substitutions altered voltage-dependence or kinetics of activation, affected inactivation (including recovery), affected the late component of *I*_Na_, abolished the current completely, or any of the above. We then evaluated the power of machine-learning techniques such as Naive Bayes (used in PolyPhen^16^), support vector machines (used in SNPs&Go^17^), and neural networks (used in SNAP^18^) to predict the outcomes from the features. An overview of our methods is shown in Fig. 1.

## Results

**Figure 2:**
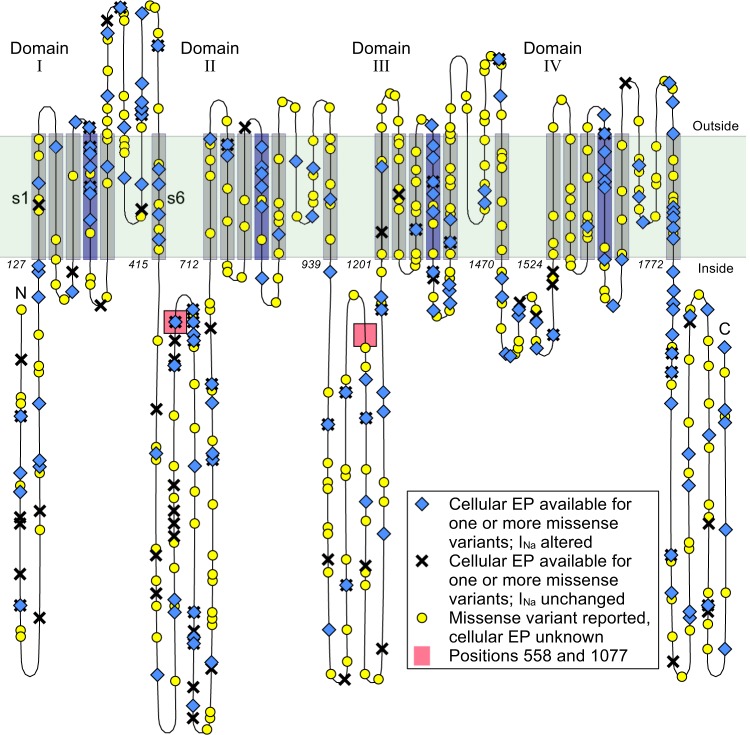
A schematic overview of Na_v_1.5^19^. The transmembrane segments are shown for all four domains. In each domain, the voltage-sensing 4th segment is coloured dark blue. The locations of the common polymorphisms H558R and del1077Q are indicated with red boxes. To show the relative size of the linkers and transmembrane segments, the diagram was constructed with near-equal spacing between residues. The linker between segments 5 and 6 of each domain is known to fold back into the membrane, as is shown in the diagram. Note that the folding of segments 5 and 6 and the inter-domain linkers in this diagram is purely symbolic, and the exact location of variants may differ. The division of *SCN5A* into domains, segments and linkers is based on the annotations given on http://www.ncbi.nlm.nih.gov.

Figure 2 Shows a schematic overview of Na_v_1.5^19^, with its four domains, each featuring six transmembrane segments (shaded regions). The fourth segment in each domain is sensitive to changes in voltage, and the linker between segments 5 and 6 is known to fold back into the membrane to create the channel pore^20^. Both the C-terminus and the linker between domains III and IV have been suggested to be involved in inactivation and the late component of *I*_Na_^21^. *SCN5A* mutations are found throughout the gene, and mutations have been reported in the clinical/biophysical literature for every transmembrane segment and every linker in the protein. By far most studies report a significant change in *I*_Na_ (Fig. 2). Whereas many studies focus on the key regions mentioned above, many variants outside of these sensitive areas have also been linked to arrhythmia, and conversely many mutations inside sensitive areas do not significantly alter *I*_Na_. A numerical view of the data is shown in Table 1 and a further breakdown of changed/unchanged EP per area is given in section 6 of the Supplement.

**Table 1:** The number of variants in our database for different categories and the number of positions on the gene at which variants occur[19]. Between brackets: the percentage of variants/positions relative to the total number possible/present.

	Variants	Positions
Total found	610/11923 (5.1%)	482/2016 (24%)
With EP data	243/11923 (2.0%)	199/2016 (9.9%)
EP changed	175	143
EP unchanged	68	64
Activation changed	69	60
Inactivation changed	125	104
Late component changed	40	35
Zero current	30	27
Total possible (with duplicates)	13357	2016
Total possible	11923	2016

We gathered 378 reports of EP-data measurements^19^. As several studies investigated the same variants (either confirming previous work or addressing new aspects), we found cellular EP for 243 variants. The final row in Table 1 gives the total number of possible amino-acid substitutions arising from a single nucleotide change in *SCN5A* before and after removing duplicate gene products. This was calculated by listing all nucleotide substitutions in the coding region of *SCN5A* and noting which ones resulted in an amino-acid switch (not including stop codons). From this we calculated that there are approximately 11923/2016 ≈ 5.91 possible mutations per position in the gene, so that while Fig. 2 accurately reflects the positions at which variants are known, it should not be taken as an indication of the coverage of our dataset, which it overstates by about six times. A list of variants and the resulting changes in cellular EP is given in section 11 of the Supplement.

### Positional properties

**Figure 3:**
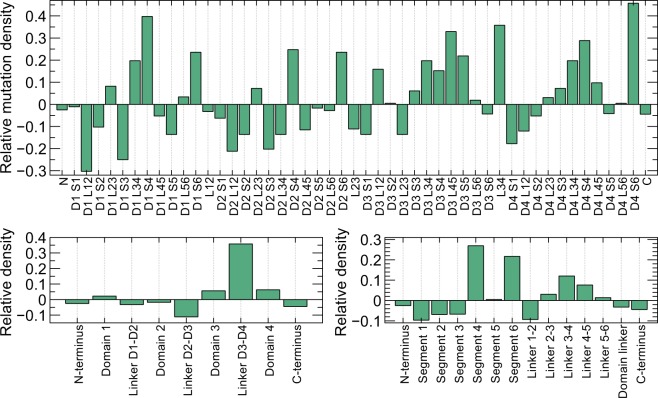
Relative mutation densities in different regions of *SCN5A*^19^. Positive values indicate a higher-than-average density of mutations in a region, while negative values indicate a relative paucity. *(Top)* Relative density for all regions of *SCN5A*, starting with the N-terminus (N), then segment 1 of domain 1 (D1S1), the linker between segments 1 and 2 in domain 1 (D1L12), etc. The linker between domains 1 and 2 is indicated as L12. *(Bottom left)* Relative mutation density in the four domains, the terminals and the domain linkers. *(Bottom right)* Density in the different regions for all domains: first segment, second segment, linker between segments 1 and 2 and so on.

We next investigated the estimated *relative mutation densities* in different regions of the channel^19^ (Fig. 3, details of how this was calculated can be found in the Methods section). The variants used for this figure were all reported in the literature on cardiac arrhythmias and cardiomyopathies (see the section “Finding mutations in the literature”). The voltage-sensing fourth segment was found to have the highest relative mutation density. Although many variants were reported in the C- and N-terminus, their large size resulted in a low mutation density. Differences between the same regions in different domains were found. For example, the linker between segments 3 and 4 was found to have a higher-than-average mutation density in domains 1, 3 and 4 but a lower-than-average density in domain 2. Comparing domains revealed similar differences, with variants in domain 2 being relatively uncommonly reported while domains 3 and 4 were most likely to contain variants reported in the scientific literature. Due to its short length, and perhaps its role in inactivation, the linker between domains 3 and 4 was found to have a very high mutation density.

**Figure 4:**
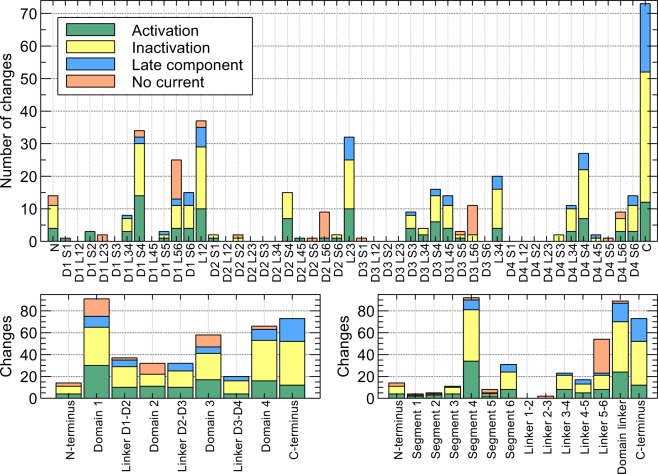
Number of times a significant change in cellular EP was reported due to variants in different regions of *SCN5A*^19^. This figure is based entirely on variants for which cellular EP data is known. Variants that induced multiple changes were counted twice, so if a variant significantly influenced both activation and inactivation the counts for both were increased by one. Conflicts in EP data were resolved by tallying votes for and against significant change (see “Machine-learning datasets”).

These results all indicate that there is a relationship between position on the gene and function, although this relationship may not be straightforward. To further investigate these structure-function relationships, we plotted the number and type of reported changes to *I*_Na_ in Fig. 4 Mutations leading to a channel that failed to produce any current occured predominantly in the pore-forming linker between segments 5 and 6^19^. This suggests that changes to the pore were investigated more often than mutations abolishing current through other mechanisms (e.g., extreme changes in voltage dependence or trafficking and folding defects). Among the transmembrane segments, the voltage-sensing fourth segment was the main contributor of changes in activation, but surprisingly the domain linkers were almost equally prone. However, the number of changes in activation per amino acid was much higher in the fourth segment than anywhere else in the gene. Changes to inactivation were common in the voltage-sensing segment, but also occurred frequently in the domain linkers and the C-terminus. Surprisingly, the linker between domains 3 and 4 showed very few mutations affecting inactivation, but this may be explained by its relatively small size. Most mutations affecting late *I*_Na_ were found in the domain linkers and C-terminus. In general, mutations affecting the late component of *I*_Na_ were found in regions also affecting inactivation, supporting the idea that late *I*_Na_ is connected to a failure to fully inactivate.

These results are consistent with previous reports of *I*_Na_ structure-function relationships^19^. For example, Kapa *et al*.^22^ investigated the predictive value of different *SCN5A* regions when predicting pathogenicity of long-QT syndrome. They found a strong link between long-QT pathogenicity and the transmembrane segments and the C-terminus, which agrees with our finding that these regions are linked to changes in the late component of *I*_Na_. While Fig. 4 shows a connection between the domain linkers and the late component, no such link was established in the study by Kapa *et al*., but this may be explained by our larger dataset and *SCN5A* -specific approach. Motoike *et al*.^21^ showed that inactivation and late *I*_Na_ are associated with both the C-terminus and the linker between domains 3 and 4. Our results confirm this role of the C-terminus, but indicate that while the 3-4 linker has a very high mutation density, the absolute number of mutations affecting inactivation and late *I*_Na_ is similar to the other domain linkers. Observations like these, i.e., the existence of inactivation-affecting mutations in regions not known to play a part in inactivation, could indicate a novel role for these regions. However, it is difficult to judge if their scarcity is a sign of low importance (causing them to surface in clinical investigations only rarely), of high importance (causing them to be hidden by incompatibility with life), or mere coincidence (see “Discussion”).

### Amino-acid properties

**Figure 5:**
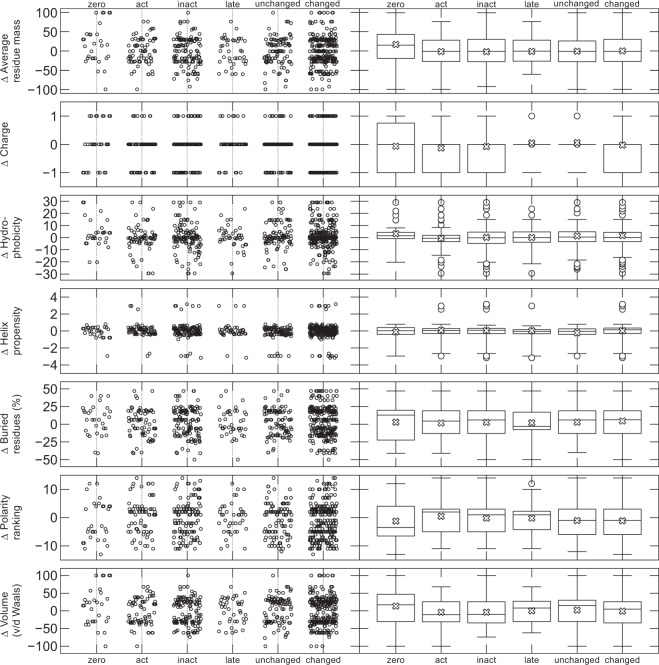
(*Left*) Changes in EP in relation to changes in amino-acid properties^19^. We calculated the difference in several properties between the new and the old amino acid involved in the substitution. From left to right, the figure shows variants that prevent all current, affect activation, affect inactivation, affect the late component, or make no significant difference to the current. The left panel shows the raw data, plotted with a slight ‘jitter’ on the x-axis to better distinguish individual points. (*Right*) Box plots for the same data. The line inside the box represents the median, and the top and bottom of the box indicate the first and third quartiles. The whiskers indicate the data within 1.5 times the inter-quartile range of the upper and lower bounds of the box, and a cross is drawn at the sample mean.

We next sought to relate the changes in EP to changes in the physico-chemical properties of the substituted amino-acids (Fig. 5). Changes in charge were only marginally more common for variants affecting activation and inactivation than for unaffected cases^19^. Large changes to *α*-helix propensity were uncommon regardless of the associated EP-change. Some structure appears due to the discrete nature of the data (there are at most 20 different values per property, corresponding to the 20 amino acids), some banding appears in the graph, notably creating gaps near zero for e.g., volume. However, no other striking patterns emerge. There were no significant differences between the groups (see section 8 of the Supplement for details).

We next addressed the question of which patterns, if any, could be found in the amino-acid substitutions of reported mutations. We calculated the ratio between observed and expected frequency of each *x* to *y* transition (where *x* and *y* represent one of the 20 amino acids)^19^. We found that certain replacements, for example arginine (R, positive charge) to histidine (H, positive charge) were seen much more often than expected, and that, in general, R was 4 times more likely to be replaced than average while tryptophan (W) was the most common substitute. However, we were unable to connect these observations to the physico-chemical properties of the amino acids. For example, charge conservation was over-represented in some cases (R to H) and under-represented in others (aspartic acid, D to glutamic acid, E). Full details are given in section 7 of the Supplement.

Taken together, the findings indicate that changes in individual amino acids are unsuitable to accurately predict the effects of an *SCN5A* variant on *I*_Na_.

### Quantitative predictions

**Figure 6:**
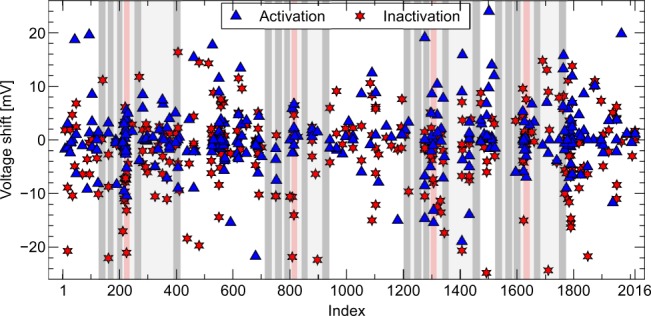
The measured shift in midpoint of activation and inactivation, plotted against the position of the amino-acid variant on the channel protein^19^. This figure was constructed from all the available EP data, and so may include the same variant more than once, if it was investigated in multiple studies. Midpoint shifts are incorporated without regards to the statistical significance of the shift. The positions corresponding to transmembrane segments are indicated using grey shading, and the voltage-sensing fourth segments are highlighted in red.

To determine if quantitative predictions about the magnitude of the EP change could be made, we investigated mutations that caused a shift in the midpoint of activation or inactivation^19^. In Fig. 6 the size of these shifts is plotted against position in the gene. Again large effects were not restricted to key areas, but observed throughout the gene. However, compared to the other transmembrane segments, the fourth segment stood out due to the large number of variants, and many large shifts were seen.

### Machine-learning results

**Figure 7:**
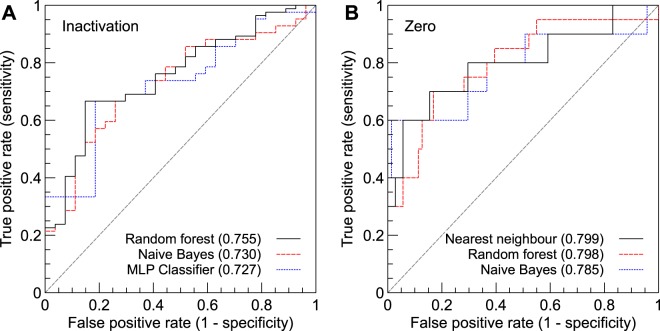
Receiver-operating characteristics on the test datasets, for the inactivation and zero-current classification problems, for the three methods that performed best on AUC. The corresponding areas (AUCs) are indicated in brackets.

Having inspected the data, we investigated whether machine-learning methods could detect structure not easily visible with the human eye. A separate dataset was created for each of the five outcomes (EP changed, activation changed, inactivation changed, late component changed, current diminished completely), creating five independent machine-learning problems. We then tested several different methods, each based on a different underlying principle, on these five problems. Table 2 shows the best results obtained for each problem, along with the method that performed best. We show accuracy (percentage of correct predictions), along with area under the receiver-operating (ROC) curve and MCC. The result from a ‘Zero-R’ classifier, which provides a baseline for comparison, are shown in brackets behind each number. ROC curves for the best-performing classifiers for the ‘inactivation’ and ‘zero current’ problems are shown in Fig. 7.

**Table 2:** Best results per problem. Zero-R classifier results are shown in brackets. The lower part of the Table shows the MCC results from Leong *et al*.^7^, for the problem of predicting pathogenicity directly.

Outcome	Accuracy	AUC	MCC	Method
Changed/Unchanged	66.7% (72.8%)	0.693 (0.5)	0.239 (0)	Naive Bayes
Activation	66.1% (62.9%)	0.657 (0.5)	0.325 (0)	Naive Bayes
Inactivation	66.7% (60.9%)	0.730 (0.5)	0.305 (0)	Naive Bayes
Late component	60.0% (65.0%)	0.530 (0.5)	0.061 (0)	k-Nearest neighbour
Zero current	91.4% (87.7%)	0.785 (0.5)	0.584 (0)	Naive Bayes
Pathogenicity			0.00 (0)	PolyPhen-2
Pathogenicity			0.00 (0)	SNPs&GO
Pathogenicity			0.15 (0)	SIFT
Pathogenicity			0.21 (0)	PROVEAN
Pathogenicity			0.24 (0)	SNAP
Pathogenicity			0.32 (0)	SNAP & Provean

For both activation and inactivation, only a modest improvement on baseline accuracy was made, but AUC and MCC could be improved considerably^19^. Predicting changes to the late component proved most difficult, with the best-performing classifier showing an MCC of only 0.061. The best results were obtained for the ‘zero current’ problem, although only a small number of variants abolished *I*_Na_ completely, leading to a very high baseline (Zero-R) accuracy of 87.7%. Predicting if the current produced *any* change in *I*_Na_ proved more difficult: although an MCC of 0.239 was reached this required sacrificing predictive accuracy, which was below the Zero-R baseline. This might be explained by the fact that the changed/unchanged problem encompasses the other four problems, and is therefore potentially more difficult.

For comparison, we also included the MCCs given by Leong *et al*.^7^ for direct pathogenicity prediction using five common methods, as well as the best-performing combination of those five. Interestingly, our changed/unchanged predictions showed a similar MCC to the more established tool SNAP, and outperformed the other methods (except for the combined SNAP & Provean meta-classifier). The ‘zero current’ problem (which can have a severe clinical phenotype) could be predicted with a higher MCC than any existing tool or combination of tools. These results suggest that some changes to *I*_Na_ can be predicted more reliably than pathogenicity.

We next ranked the different features (variant properties) from the highest to the lowest information gain^19,23^. For the inactivation problem, the most useful properties were region type (N-terminus, segment 1, linker 5-6, etc.), the distance to the pore-forming linker between segments 5 and 6 (where distance was measured by counting the number of residues on the protein), the distance to segment 5 and the distance to the linker between domains 3 and 4. Region type was the most useful feature for all four problems, and only the zero-current dataset assigned a non-zero information gain to any of the physical amino-acid properties. Even difference in charge resulted in a very small information gain in the voltage-dependent activation problem, which is consistent with the findings in Fig. 5. The information-gain rankings for each problem are given in section 9 of the Supplement.

Finally, we assessed the performance of the same methods on the problem of predicting discretised shifts in activation and inactivation (e.g., predicting if the shift in midpoint of activation was more positive than 3 mV, more negative than −3 mV, or between −3 mV and 3 mV)^19^. The results for inactivation are shown in Table 3. Here, the Random forest method performed best, but overall accuracy was low. Similar results were obtained for activation (not shown).

**Table 3:** Success rate for predicting discretised shifts in midpoint of inactivation. Shifts were grouped into three categories: more negative than −3 mV, between −3 mV and 3 mV, or more positive than 3 mV.

Method	Accuracy	AUC
Zero-R	43.8%	0.5
Random forest	46.6%	0.640
Naive Bayes	46.6%	0.625
MLP classifier	49.3%	0.606
Support-vector machine	50.7%	0.617
k-Nearest neighbour	45.2%	0.626

## Discussion

Predictors of *SCN5A* variant pathogenicity suffer from poor performance. We hypothesised that taking a smaller leap, from *SCN5A* variants to changes in *I*_Na_, would reduce the complexity of this problem and lead to improved predictions. To test this, we assembled a large dataset containing information about non-synonymous *SCN5A* missense mutations reported in the literature on channelopathies and cardiomyopathies, and the resulting changes to *I*_Na_^19^.

We found that missense mutations occur throughout the gene, but with a higher rate per amino acid in transmembrane segments 4 and 6 than in any other segment^19^. The linkers between domain 1-2 and 2-3 showed a lower than average mutation density, but a high density was seen in the linker between domains 3 and 4. Of the four domains, domain 2 had the lowest mutation density.

Changes in function correlated with the location of the variant on the gene^19^. Complete absence of current was often observed for variants in the pore-forming linkers between segment 5 and 6. Effects on inactivation were mainly seen in the voltage-sensing 4th segment, the 6th segment, the domain linkers and the C-terminus. This is consistent with earlier findings by Brunklaus *et al*.^8^, who showed variants in these regions were often implicated in long-QT syndrome type 3. Mutations influencing late *I*_Na_ often occurred in the C-terminus, and appeared mostly in areas also affecting inactivation.

The reported amino-acid substitutions did not occur with the frequencies that would be expected based on unbiased single nucleotide changes^19^. Instead, arginine substitutions were strongly overrepresented in our dataset, while tryptophan was the most common substitute residue. However, no strong relationship between the change in amino-acid properties and the variant’s electrophysiological effects could be seen.

We applied five different machine-learning techniques to the problems of predicting qualitative changes in EP^19^. In terms of accuracy, no major improvements over baseline (i.e., the performance of a Zero-R classifier) were seen. However, better-than-chance AUCs and MCCs were obtained. The region type (see “Machine-learning features”) was found to be the most informative feature of a variant in all four problems examined. Again, the physical properties of the exchanged amino acids provided little information.

By comparing our results to previous work by Leong *et al*.^7^, we found that full reduction of current and changes to activation and inactivation can be predicted with higher MCCs than achieved by direct predictors of pathogenicity. This supports our hypothesis that *I*_Na_ changes can be predicted more reliably than pathogenicity. However, the best combination of tools (SNAP & PROVEAN) achieved comparable results, so the difference is not as great as might have been expected. Overall, the performance of all methods was good in terms of AUC and MCC, but still failed to deliver the accuracy needed to create a two-stage *in-silico* predictor. This suggests that fully *in-silico* predictions of unclassified *SCN5A* variant pathogenicity, either direct or indirect, are still out of reach. Instead, our results underscore the need for functional *in-vitro* studies to assess the risks posed by *SCN5A* variants.

The predictive power of a dataset depends on three critical factors: it must contain enough information, it must be unbiased (balanced), representing equally plausible outcomes in equal measures, and it must be *internally consistent*^19^. Once a dataset meeting these criteria is available, it must be reformatted in such a way that each variant is represented by appropriate features. These are properties that are indicative of some essential aspect of the substitution, such as its location in the protein or the associated change in electrical charge. The information gain per feature (shown in section 9 of the Supplement) was low or zero for many of the selected features, indicating that this is an area where improvements can be made.

Considering the work that goes into measuring the cellular EP changes due to a mutation, the total number of 378 EP recordings of 243 unique variants is an impressive achievement by the collective scientific community. As shown in section 3 of the Supplement, the number of EP data reports for *SCN5A* variants has steadily increased each year^19^. One development that could lead to dramatic increases in data is the inclusion of *paralogues*^24,25^. This entails aligning the sequence of *SCN5A* with that of highly similar sodium channel genes (i.e., *SCN1A*, *SCN2A*, *SCN3A*, etc) and using data about their variants for similar positions in *SCN5A*. Besides increasing the size of the dataset, care must be taken to improve the manner in which experimental conditions are reported, perhaps using a scheme such as outlined by Quinn *et al*.^26^. For example, while variations in the *α*-subunit are known to affect *I*_Na_ properties^27^, and while sequencing methods and unique clone IDs are now readily available, a significant fraction of experimental reports still do not state the exact *α*-subunit used (see section 4 of the Supplement for details). Our approach therefore necessarily mixes data from different *α*-subunit varieties. If future experiments all include the most common Q1077del isoform and provide accession numbers for each subunit they use, this limitation can be overcome.

Almost all of the variants listed in our database were reported as a result of a clinical investigation of patients with some pathological cardiac phenotype; accordingly only 28% of EP-data results in our dataset showed no change. Indeed, the variants we report as ‘unchanged’ were almost exclusively published in papers reporting that an effect *was* seen in the presence of a secondary factor (for example the common polymorphism H558R or drugs such as lidocaine)^19^. Imbalanced datasets, such as the ones considered in the present study, pose well-known difficulties for machine-learning algorithms; as they will be biased towards the majority group^28^. In agreement, the least biased dataset in our study (zero current predictions) showed the highest AUC and MCC, underscoring the need to improve balance in the dataset and further reduce this reporting bias. The lack of balance in our datasets may also explain why the simpler machine-learning methods (Naive Bayes, Nearest neighbour) outperform more complex methods (e.g., support-vector machines and the MLP classifier), as their more limited ability to fit to complex patterns in the training data can help prevent overfitting.

Compatibility with life provides a more subtle bias: since mutations that prevent all current (such as frameshift mutations or trafficking defects) are still commonly reported, it seems that if a heterozygous mutation renders a channel completely ineffective, the body can compensate by upregulating the overall production of cardiac sodium channels or downregulating others^29^. By contrast, gain-of-function mutations that seem less extreme from a biophysical perspective (e.g., ones that cause a strong increase in late *I*_Na_ or a large leftward shift in voltage-dependence, but do not fully prevent any current) may be incompatible with life even heterozygously, causing them to be under-represented in our dataset. There is also a chance that a non-functioning heterozygously expressed channel interferes with the unmutated channel via dominant-negative *α*-*α*-subunit interaction^19,30^.

Addressing the problem of dataset bias requires a targeted approach^19^. Using Fig. 3 and the amino acid substitution rates given in section 7 of the Supplement as a guide, variants in under-represented areas or with uncommon amino-acid substitutions could be identified and investigated. This would involve investing resources into variants with no clear clinical significance, but would greatly increase the value of the combined EP data gathered so far. A cheaper alternative may be to put out a call to all labs harbouring unpublished negative data to process and publish it, ideally in a freely accessible online database.

In our dataset, more than one EP data report exists for 74 variants, and some inconsistencies can be seen for 43 of those^19^ (58%; see section 5 of the Supplement for two examples). For figures such as Fig. 4, which dealt with EP data *classes* (activation, inactivation, etc.) we used a voting system to resolve conflicts (see “Electrophysiological characterizations”). For numerical data (midpoint shifts) we used a filtered version of the dataset in which conflicts were resolved by selecting the favoured experimental conditions. In the future, when even more data is available, it may be possible to select only a subset of the data that conforms to a certain experimental set-up. This will resolve the issue of different experimental conditions, but the best way to deal with remaining conflicts is an open issue.

Creating a workable predictor will require finding features that capture the difference in physical properties before and after an amino-acid substitution. The current set of features was chosen based on mechanistic reasoning (e.g., the location and physico-chemical properties of the amino acids can be presumed to affect channel function), and ideas from the literature (e.g., inclusion in or proximity to the voltage sensor is often quoted as a reason that a mutation could be important to channel function). However, the current list is not exhaustive, and the information-gain figures in section 9 of the Supplement further show that not all features are useful for classification. Hence, the need for more descriptive features is a clear outcome of this first study. One approach could be to build a full physical model and use molecular-dynamics methods to compare channel structure before and after a mutation^19^. However, such models are usually based on homology models that do not include the important domain linking segments and terminals, and molecular-dynamics simulations are computationally very expensive. An alternative approach may be to segment the gene, for example using the ‘basic units of protein structure’ proposed by Berezovsky *et al*.^31^, and then to inspect the amino-acid properties on a per-segment basis or via the calculation of three-dimensional ‘moments’^32^.

Finally, it will also be important to take into account any post-translational modification of channel function via the interaction with various signalling mechanisms^19,33^.

In conclusion, the effects of missense mutations in *SCN5A* on *I*_Na_ are difficult to predict using machine-learning methods. However, our first attempts at solving this problem already provide more reliable predictions than established methods for solving the more complex problem of predicting *pathogenicity*. Our approach can be further improved by expanding the size of the dataset, reducing its reporting bias, and finding features that better characterise the effects of a mutation on Na_v_1.5 structure. These results underscore the need for functional studies, for example *in-vivo* experiments or cellular expression systems combined with computational modelling, when assessing the impact of mutations in *SCN5A*.

## Methods

### Finding mutations in the literature

To find publications on *SCN5A* missense mutations, we scanned through all PubMed results for the term ‘ *SCN5A* mutation’^19^. In addition, we screened previously published overview papers^3–5,34–37^. From the selected papers, we listed all nonsynonymous missense mutations for which cellular EP data was known, or which were associated with a pathological clinical phenotype. Because we focused on changes to channel function, we only noted amino-acid substitutions, without investigating the underlying nucleotide change. Common polymorphisms such as H558R were treated as variants, and included in the database as such. Reports investigating variants in the presence of H558R were treated as double variants, and excluded from our database.

### Numbering of amino acids

In numbering the mutations, we used the position on the full, 2016 residue long sequence for human *SCN5A* (Isoform 1, GenBank accession number AC137587). In some cases this meant using a different numbering than in the original source^19^.

### Electrophysiological characterizations

Where possible, we collected information about the changes to whole-cell *I*_Na_ resulting from each mutation^19^. First, we asked whether or not the mutated channel conducted a current large enough to measure (making no distinction between blocked pores and other factors such as trafficking defects). For conducting channels, we then looked if activation, inactivation (including recovery), or the late component were affected. Each of these fields was recorded as ‘measured and affected’, ‘measured and not affected’, or ‘not measured’. The distinction between affected and not affected was made based on the significance (p-value) given in the paper and the wording used by the authors. Only measurements from homozygous mutant expression were included. Where possible, we calculated shifts in midpoint of activation and inactivation from the data given in the paper.

### Experimental set-up

Every measurement recorded in the EP database was annotated with three fields: the type of *α*-subunit, the cell-type, and the presence of *β*_1_-subunits^19^. In order of descending frequency the cell types were HEK (HEK293 and tsA-201), *Xenopus laevis* oocytes and CHO, but we also found a small number of measurements in COS cells and transgenic mouse myocytes. Some papers failed to mention the cell type and were listed as ‘unknown’.

Many papers did not give precise information about the type of *α*-subunit used^19^. We found at least five different subunits. Some investigators used a construct corresponding directly to either isoform 1 or isoform 2. Isoform 1 was denoted as *a*; it has a glutamine at position 1077 (Q1077) and is 2016 amino acids long (GenBank accession numbers AC137587 and NM_198056). Isoform 2 was denoted *b*; it lacks the 1077 glutamine (Q1077del) making it 2015 amino acids long (GenBank accession numbers AY148488 and NM_000335). Isoform 2 is now held to be the most common isoform, and most often used in electrophysiological investigations^27,38^. A number of *α*-subunit clones have historically been used, which turned out to contain unintended variants. The most common of these is often called ‘hH1’ (GenBank accession number M77235) and is equal to isoform 1 except for the rare variant R1027Q. We labelled this *α*-subunit a*. All papers mentioning ‘hH1’ as the only reference were recorded as using a*. A less common variant corresponding to isoform 2 with a T559A substitution was labelled b*. Finally, some of the earliest papers had isoforms that turned out to contain additional rare and/or common variants, and in many cases the exact *α*-subunit type was not mentioned in the paper. An overview of the four main *α*-subunit types found is given in section 1 of the Supplement.

### Relative mutation densities

To see if the mutations in our dataset were clustered in specific regions, we calculated mutation densities, as the *number of mutations in the region* divided by length of the region. Here, the number of mutations was counted as the number of unique reported amino-acid substitutions, and the lengths were measured as the number of amino acids in a region. We then calculated the same measure for the entire gene. By subtracting this gene-wide density from the regional densities, we arrived at relative mutation densities per region. For this regional measure, a positive value indicates there are more mutations in the region than in the overal gene, while a negative value indicates relatively fewer mutations were reported in the region.

### Machine-learning datasets

We created two types of datasets used to test the power of machine-learning algorithms to predict cellular EP^19^. Both contained a number of features describing each mutation (see below). In addition, each dataset contained an outcome field describing the outcome to predict. We used both qualitative and quantitative outcomes.

A dataset was created for each of the qualitative outcomes ‘changed/unchanged’, ‘activation affected’, ‘inactivation affected’, ‘late component affected’, and ‘zero current’^19^. Only variants for which the outcome was known were included. Where multiple EP recordings were available, conflicts were resolved by a “majority vote” amongst the publications. Variants with an equal number of reports claiming ‘affected’ and ‘unaffected’ were not included in the dataset (resulting in the removal of 3.4% of the data).

These qualitative datasets were then split into a ‘training’ set (66.7% of the data), and an independent ‘test’ set (33.3% of the data). The training set was used to train and tune classifiers (using 10-fold cross-validation to determine and optimise the MCC), after which predictive power was assessed by evaluating the resulting model on the test set. Further details of our tuning procedure are given in section 10 of the Supplement.

For (semi-)quantitative predictions, we created a dataset of shifts in midpoint of activation or inactivation^19^. Instead of using numerical voltages, we discretised the voltage shifts by assigning them to one of three intervals: (−∞, −3mV), [−3mV, 3mV], (3mV, ∞). Where multiple values were available, we selected a single recording by looking at the experimental conditions used. First, recordings co-expressing *β*_1_-subunits were preferred. Secondly, we looked for recordings made with the *α*-subunit ‘b’. Thirdly, we prioritised recordings in HEK cells over others (since these were the most common in our dataset). Finally, any remaining conflicts were resolved by selecting the most recent recording.

### Machine-learning features

Although a missense mutation can be uniquely described by its position on the gene and the replacement amino acid, this compact description is unlikely to provide enough information to be used for classification. We therefore created a varied set of features indicating aspects of the mutation that are likely to be important (e.g., position, physico-chemical properties), including factors that have often been cited in literature (e.g., proximity to the voltage sensing 4th segment).

First, we added features based on the annotations for *SCN5A* found on http://www.ncbi.nlm.nih.gov. These were: *side* (cytoplasmic, transmembrane, extracellular), *segment type* (terminus, segment, linker, domain linker) or *region type* (N-terminus, segment 1, segment linker 1-2, domain linker 1-2, etc. see Fig. 1). In addition, we added the distance (in amino-acid counts) of each position to selected regions of the protein (e.g., distance to the 4th segment, 5th segment, etc. see Fig. 1).

Next, we added features describing the difference in physico-chemical properties between the substituted amino acids^19^. We added the change in average residue mass^39^, percentage of buried residues^39^, van der Waals volume^39^, and polarity ranking^39^, amino-acid charge^39^, hydrophobicity ranking^40^ and *α*-helix propensity^41^. Next, we added a measure of amino-acid similarity^42^ and of substitution likelihood^43^.

Finally, we added a measure of conservedness for each position in the amino acid^19^. This was calculated by performing a sequence alignment of human sodium-channel genes and isoforms, as listed in section 2 of the Supplement. To further emphasise the functionally most important positions, two non-human sequences were added that have a history in cell electrophysiology: the eel sequence by Noda *et al*.^44^ and the sequence by Rosenthal *et al*.^45^ of *Doryteuthis opalescens*, a squid formerly known as *Loligo*. Sequences were aligned using Clustal 2.1^46^ using the ‘Gonnet-250’ scoring matrix. The resulting conservedness score for each position of *SCN5A* was then added to the set of machine-learning features.

### Machine-learning methods

Weka 3.9.2^47^ was used to apply different machine-learning methods^19^. To increase our chance of success, we employed methods based on very different underlying principles. We used a tree-based ‘Random forest’ classifier^48^, a Bayesian-statistics based ‘Naive Bayes’ classifier^49^, a classifier based on a multilayer perceptron (MLP, a type of neural network), a support-vector machine classifier^50^ and a nearest-neighbour classifier, which is an example of an instance-based learning method^51^. Each method was tuned to achieve the best MCC on a training dataset, before being evaluated on an independent test set. Details of this tuning are given in section 10 of the Supplement.

Performance was evaluated using three measures: accuracy (the percentage of correct guesses), the area under the receiver operating characteristic^52^ (or area-under-curve, AUC), and the Matthews correlation coefficient (MCC)^19^.

As a baseline for each measure a Zero-R classifier was used^19^. This simply assigns the most common class in a dataset to every new instance it evaluates. Thus, when a dataset contains 90 instances where the current is affected and 10 where it is not, the Zero-R classifier always predicts ‘affected’ and so has an accuracy of 90%. Using this baseline, another method with an apparently high accuracy of 91%, can be seen to have only made a small improvement over pure chance.

To see which features of the data were most useful for classification, we ranked them according to their *information gain*. This was calculated using Weka’s ‘InfoGainAttributeEval’ method^53^, which uses the definition given by Quinlan^23,54^.

### Other software tools

All gathered data was stored in an SQLite database, and analysis was performed using Python^19^. Statistical tests were carried out using SciPy^55^.

## Electronic supplementary material

Supplementary information

## Data Availability

The datasets generated during and/or analysed during the current study are available from the corresponding author on reasonable request. Relevant gene accession codes are given throughout the text.
