# Supplementary material for: Predicting changes to INa from missense mutations in human SCN5A
Source: Sci Rep. 2018 Aug 24;8:12797. doi: 10.1038/s41598-018-30577-5 (PMC6109095; doi:10.1038/s41598-018-30577-5)
Supplement: Supplementary file 1 — Supplementary information [file 41598_2018_30577_MOESM1_ESM.pdf]

Supplement: Predicting changes to I_{Na} from missense mutations in human *SCN5A*

Michael Clerx[1,2], Jordi Heijman[1], Pieter Collins[2], and Paul G.A. Volders[1,*]

[1]Department of Cardiology, Cardiovascular Research Institute Maastricht, Maastricht University Medical Center, Maastricht, 6202 AZ, The Netherlands
[2]BioInformatics and BioMathematics, Department of Data Science and Knowledge Engineering, Maastricht University, Maastricht, 6200 MD, The Netherlands
[*]p.volders@maastrichtuniversity.nl

Contents

1 Different α-subunit types used in investigations 2
2 Human sodium-channel isoforms 2
3 Reported mutations over time 2
4 Reporting of experimental conditions 3
5 Inconsistencies in the dataset 3
6 Changed/Unchanged EP per region 4
7 Observed and expected amino-acid substitution ratios 5
8 Physico-chemical properties per outcome 8
9 Information gain per feature 9
10 Tuning the classifiers 11
11 EP data overview 14
References 22

1 Different α-subunit types used in investigations

Table 1 shows the different α-subunit types used in I_{Na} experiments that we encountered in our literature search. The code used in the paper for each type is given, as well as a description and, where possible, a GenBank accession number.

Code	Description	Acc. No.
a	Isoform a (Q1077)	AC137587
b	Isoform b (Q1077del)	AY148488
a*	hH1 (Q1077; R1027Q)	M77235
b*	hH1a (T559A; Q1077del)	None

Table 1. α-subunit types of the I_{Na} channel.

2 Human sodium-channel isoforms

Table 2 shows the different human sodium-channel isoforms and their accession numbers.

Name	Accession number	Name	Accession number
SCN1A, isoform 1	NP_001189364.1	*SCN5A*, isoform 3	NP_001092874.1
SCN1A, isoform 2	NP_008851.3	*SCN5A*, isoform 4	NP_001092875.1
SCN1A, isoform 3	NP_001159436.1	*SCN5A*, isoform 5	NP_001153632.1
SCN2A, isoform 1	NP_001035232.1	*SCN5A*, isoform 6	NP_001153633.1
SCN2A, isoform 2	NP_001035233.1	*SCN7A*	NP_002967.2
SCN3A, isoform 1	NP_008853.3	*SCN8A*, isoform 1	NP_055006.1
SCN3A, isoform 2	NP_001075145.1	*SCN8A*, isoform 2	NP_001171455.1
SCN3A, isoform 3	NP_001075146.1	*SCN9A*	NP_002968.1
SCN4A	NP_000325.4	*SCN10A*	NP_006505.2
SCN5A, isoform 1	NP_932173.1	*SCN11A*	NP_001274152.1
SCN5A, isoform 2	NP_000326.2		

Table 2. All used human sodium-channel isoforms.

3 Reported mutations over time

Fig. 1 shows that the number of publications mentioning mutations in *SCN5A* has been steadily increasing each year, while the number of newly reported mutations exhibits more fluctuation. The number of EP data reports has also seen a gradual increase over time.

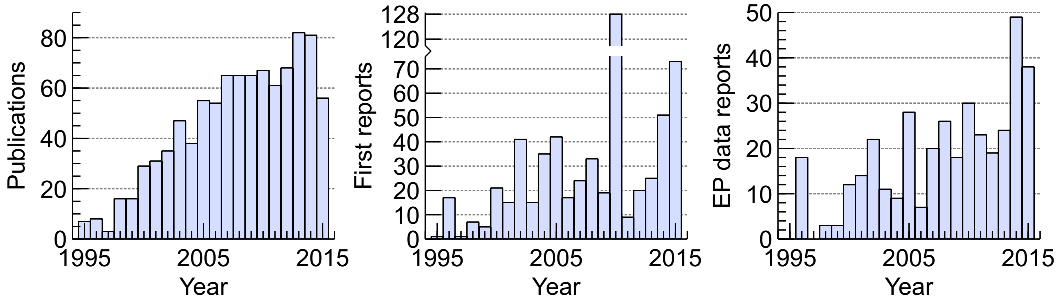

Figure 1. (*Left*) The number of publications on PubMed matching the query '*SCN5A* mutation', per year. (*Centre*) The number of first reports of mutations in our database, per year. Note the broken y-axis to accommodate the large number of mutations reported in 2010. (*Right*) The number of EP data reports in our database, per year.

4 Reporting of experimental conditions

Fig. 2 shows that, despite the availability of sequencing and databases for genes and gene products, the number of publications not reporting the exact α-subunit used is relatively high.

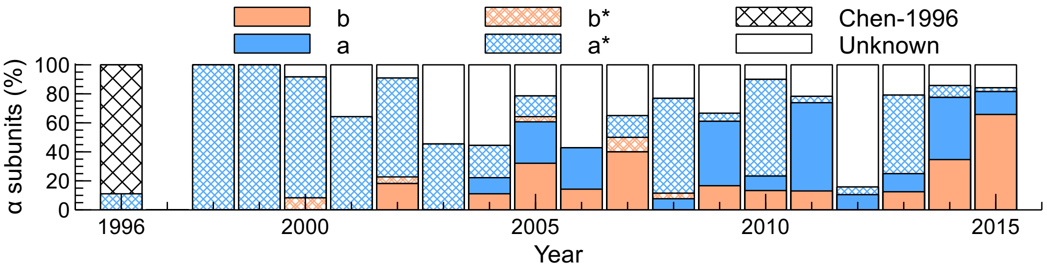

Figure 2. The different α-subunits used, in percentages per year. The fraction of subunits with some uncommon variant (a*, b*) can be seen to diminish over time, but the proportion of studies that do not report the variant they use is still significant.

5 Inconsistencies in the dataset

Two examples of mutations reported multiple times are given in Table 3. The first, R222Q, was investigated using different α-subunits, with and without β_1-subunits and in different expression systems but all experimenters obtained similar results. The second, D1790G, was investigated under more similar conditions, but experimenters found different results.

**R222Q**						
**A**	**I**	**L**	α	β_1	**Cell**	**Reference**
yes (6)	yes (9)	no (8)	a	no	HEK	Cheng et al. 2010[1]
yes (13)	yes (13)	no (10)	b	no	HEK	Cheng et al. 2010[1]
yes (11)	yes (10)		a	yes	COS	Laurent et al. 2012[2]
yes (10)	yes (10)			yes	CHO	Mann et al. 2012[3]
yes (8)	yes (8)	no (-)	a	no	CHO	Nair et al. 2012[4]
yes (13)	yes (14)	no (7)		yes	HEK	Beckermann et al. 2014[5]
**D1790G**						
**A**	**I**	**L**	α	β_1	**Cell**	**Reference**
no (-)	no (9)		a*	no	HEK	An et al. 1998[6]
no (6)	yes (20)	no (20)	a*	yes	HEK	An et al. 1998[6]
	yes (4)		a*	yes	HEK	Abriel et al. 2000[7]
yes (9)	yes (9)	yes (-)	a*	yes	HEK	Baroudi et al. 2000[8]
yes (6)	yes (13)	no (-)	a*	no	HEK	Wehrens et al. 2000[9]
no (6)	yes (6)		a*	yes	HEK	Liu et al. 2002[10]
yes (8)	yes (16)			no	HEK	Liu et al. 2003[11]

Table 3. Consistent and inconsistent EP-change reports. Mutations can have an effect on activation (A), inactivation (I) and late I_{Na} (L). Changes are characterized as yes/no or unmeasured, and the number of cells used to test the mutant are shown in brackets. In addition, the used expression system is shown, along with the α-subunit used, the co-expression of β_1-subunits, and the original reference.

6 Changed/Unchanged EP per region

Fig. 3 shows the number of mutations reported to affect or not affect the EP, for different regions of *SCN5A*. The domain linkers show many mutations that do not affect EP, but no-effect mutations can also be found in areas more strongly associated with channel function, such as the voltage-sensing segment 4, the pore-forming linker between segments 5 and 6, and the C-terminus which has frequently been implicated in inactivation defects.

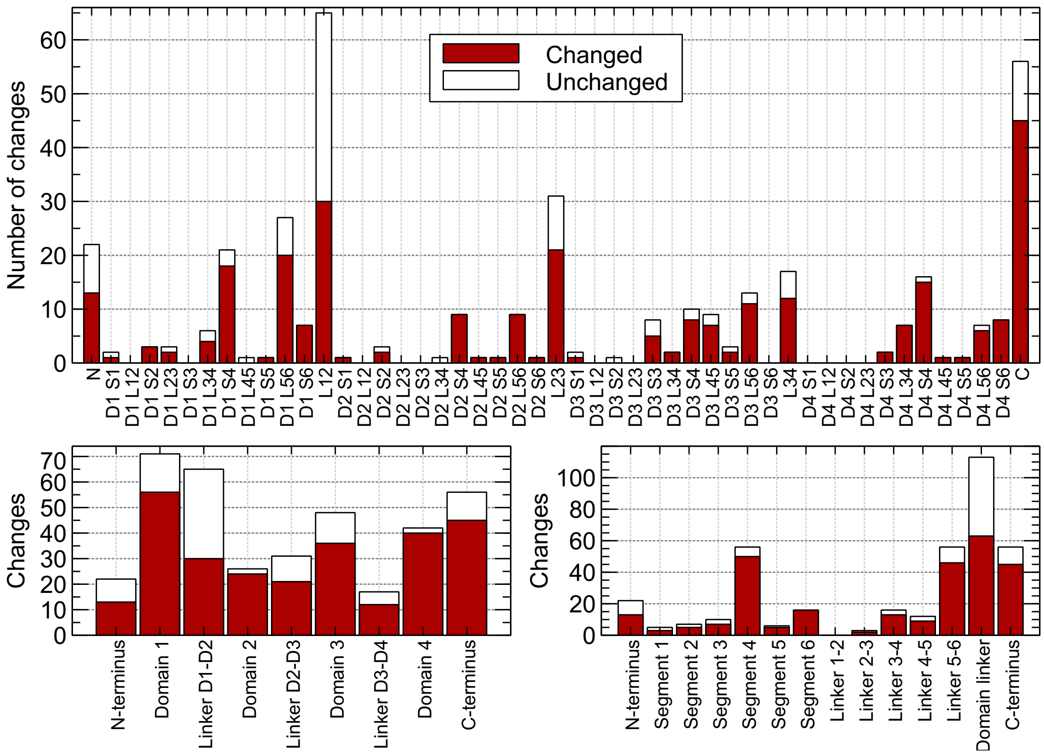

Figure 3. The number of reports describing changed or unchanged EP for different regions of the gene. Conflicts in EP data were resolved by tallying votes for and against significant change (see "Machine-learning datasets" in the main text).

7 Observed and expected amino-acid substitution ratios

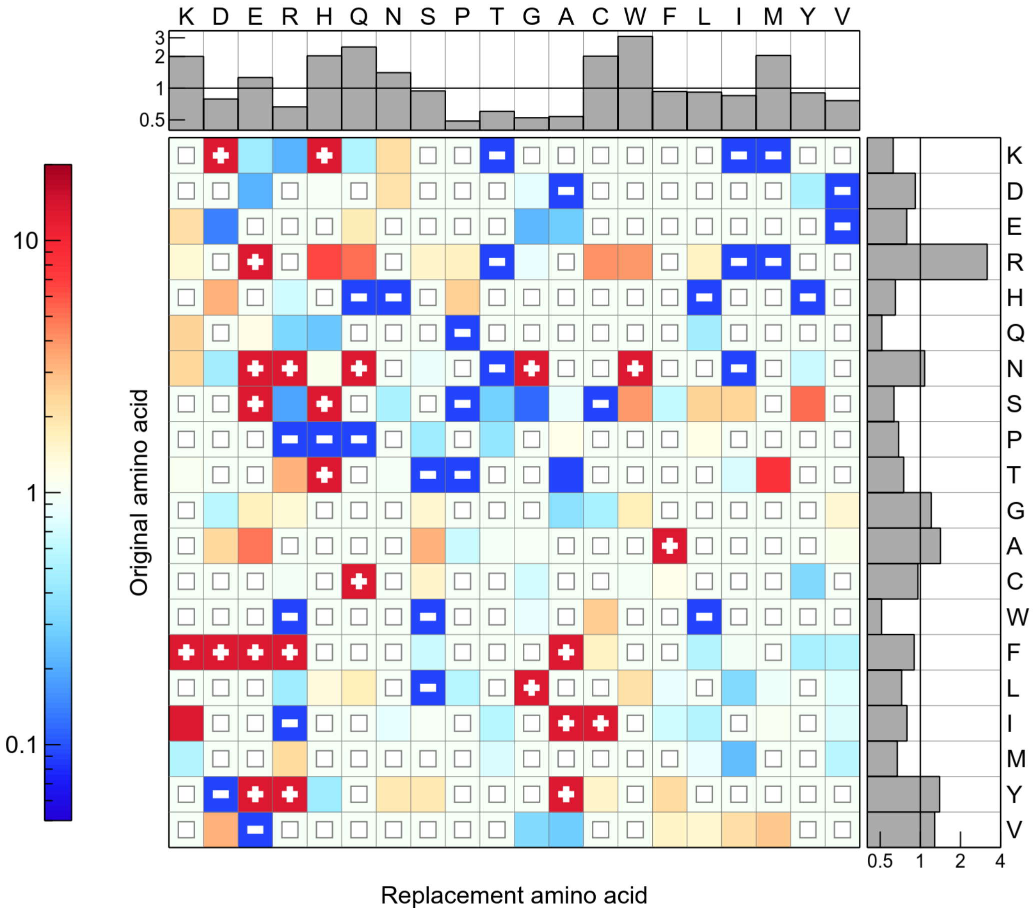

Figure 4. Central panel, the ratio of found amino-acid substitutions versus expected substitutions. Each row represents the original amino acid, and each column the residue it was replaced with. A dark red square indicates this substitution happened more often than expected, while a dark blue square shows that the substitution happened fewer times than expected. White squares indicate substitutions that were neither observed nor expected, while plus symbols (+) indicate substitutions that are present in the dataset, but cannot result from a single nucleotide switch. Minus signs (-) indicate amino-acid substitutions that could occur, but were not observed in our data. The cumulative ratios for the old and the new amino acids are shown in the right and top panels, respectively. Details of how the figure was constructed are given below.

In Fig. 4, a colour-coded representation of the amino-acid substitutions in our database is shown. Each square in the central panel shows if a particular amino-acid substitution occurred more frequently or less frequently than expected. The histograms at the top and side show the over/under-representation of amino acids in general. R was 4 times more likely to be replaced than average, and tryptophan (W) was the most common substitute. Interestingly, the results were difficult to explain using amino-acid properties. For example, charge conservation was over-represented in some cases (R to H) and under-represented in others (aspartic acid, D to glutamic acid, E).

7.1 Calculating over/under-representation of amino-acid substitutions

The figure above was created to investigate whether amino-acid substitutions in our data set occur with the frequency that could be expected if it they resulted from a random process. The steps in the calculation are detailed below.

7.1.1 Observed amino-acid substitutions

We can count the number of amino acid $x \to y$ substitutions in our data set, and call it $N^o_{x\to y}$. (Note that when counting, we count each unique mutation once, regardless of the number of reports.) From this, we can calculate ratios:

$$r^o_{x\to y} = \frac{N^o_{x\to y}}{\sum_{i,j} N^o_{i\to j}}$$

where the sum is over all observed substitutions $i \to j$. In this notation, $r^o_{x\to y}$ gives the ratio of x to y amino-acid substitution in the data set, and $\sum_{i,j} r^o_{i\to j} = 1$.

7.1.2 Predicted amino-acid substitutions

To see if there is anything unusual about our observed substitutions, we want to compare them with the expected ratios. To calculate these, we perform the following steps:

1. List all the nucleotides in *SCN5A* (we have 2016 amino acids, each encoded by a triplet of nucleotides, so 6048 nucleotides in the coding region).
2. List all possible (non-synonymous) nucleotide substitutions (there are 4 possible nucleotides, so 3 possible substitutions, for a total of 18,114 possible nucleotide substitutions).
3. Assign each of these substitutions a weight (e.g., a weight of 1 if we regard all substitutions as equally likely).
4. For each possible substitution, use the genetic code to work out the resulting amino-acid substitution.
5. Discard all nucleotide substitutions that do not change the amino-acid sequence, or that result in a premature stop codon, i.e., remove all synonymous and nonsense mutations to leave only missense mutations.
6. At this point we have a list of nucleotide substitutions as pairs (position, new nucleotide). For each pair we also know a weight and the resulting amino-acid substitution (position, new amino acid). Note that the position for nucleotides ranges from 1 to 6048, while position for amino acids is in the range 1 to 2016 (=6048 / 3). Because different nucleotide triples can produce the same amino acid, some amino-acid substitutions appear multiple times in this list.
7. In the next step we create a unique list of amino-acid substitutions by grouping together any nucleotide substitutions with the same amino-acid result and summing the weights in each group.
8. We now have a list of unique, weighted amino-acid substitutions. In the next step, we discard the position information, creating a list of (old acid, new acid) pairs, again summing the weights.
9. We can now write the weight of each x to y amino-acid substitution in this data set as $W^e_{x\to y}$ where each $W^e_{x\to y}$ is proportional to the expected number of observations of the $x \to y$ substitution.
10. Finally, we calculate the ratio
$$r^e_{x\to y} = \frac{W^e_{x\to y}}{\sum_{i,j} W^e_{i\to j}}$$

For *SCN5A*, we find a total of 18,144 nucleotide substitutions including missense and nonsense mutations. In this set, we have weights ranging from 5.355 to 50.85, with mean and sample standard deviations of 17.5 and 12.8. When we remove missense and nonsense mutations this leaves 13,357 substitutions, some of which result in the same amino-acid sequence, so that we find 11,923 amino-acid substitutions (position, old amino acid, new amino acid). In this set, the minimum weight is 5.355, the maximum weight is 64.635, and the mean and sample standard deviation are 18.4 and 13.3 respectively.

7.1.3 Observed v.s. expected

We can now calculate the quantity of interest; the ratio of observed versus expected, as

$$r_{x\to y} = \frac{r^o_{x\to y}}{r^e_{x\to y}}$$

For this measure, values greater than 1 indicate over-abundance, while values less than 1 indicate fewer observerations than expected.

One issue remains: how to deal with zeroes in this equation? As this is a ratio, ideally we would plot it on a logarithmic axis. For this, both $r^o_{x\to y} = 0$ and $r^e_{x\to y} = 0$ are problematic cases. To handle these cases, we used the imputations given below:

	**Expected**	**Not expected**
**Observed**	$r_{x\to y}$	$r_{\max}$
**Not observed**	$r_{\min}$	1

Table 4. Nucleotide substitution weights

Here $r_{\min} = \min r_{x\to y}$ and $r_{\max} = \max r_{x\to y}$ are the minimum and maximum values of $r_{x\to y}$ for the mutations that were both expected and observed.

7.1.4 Marginals

We can define marginals as

$$r_{x\to} = \frac{r^o_{x\to}}{r^e_{x\to}} = \frac{\sum_y r^o_{x\to y}}{\sum_y r^e_{x\to y}}$$

and

$$r_{\to y} = \frac{r^o_{\to y}}{r^e_{\to y}} = \frac{\sum_x r^o_{x\to y}}{\sum_x r^e_{x\to y}}$$

Here $r_{x\to}$ gives the ratio of observed v.s. expected mutations away from x, while $r_{\to x}$ gives the observed/expected ratio of mutations to x.

7.1.5 Weighting

Not all nucleotide substitutions occur in the with the same frequency in the human genome. For example, transitions (A/G and C/T) are more common than transversions (A/C, A/T, C/A, C/G). In addition, the neighbouring nucleotides influence the ratio with which a substitution is observed[12]. We calculated weights for each possible amino-acid substitution in *SCN5A* using data from ref[13].

**Substitution**	**Weight**
A → C, C → A	9.0
A → G, G → A	32.8
A → T, T → A	7.5
C → G, G → C	8.9
C → T, T → C	32.8
G → T, T → G	9.0

Table 5. Nucleotide substitution weights

Next, to include neighbouring nucleotide effects, we modified the weights using the following multiplication factors (again, based on ref[13], table 1).

Context	Multiplier
A on 5’ side	1.05
C on 5’ side	1.24
G on 5’ side	0.92
T on 5’ side	0.84
A on 3’ side	0.85
C on 3’ side	0.92
G on 3’ side	1.25
T on 3’ side	1.03

Table 6. Weight multipliers for nucleotides on 5’ or 3’ side

8 Physico-chemical properties per outcome

In Figure 6 of the paper, we looked at the difference in physico-chemical properties of the amino acids involved in a substitution, and grouped these per outcome. For each property, we then used a Kruskal-Wallis test to test the null hypothesis that each group (outcome) was from the same distribution. Because the ‘changed’ group contains all samples in the ‘zero’, ‘act’, ‘inact’, and ‘late’ groups, we did not include this group in the test.

Property	H statistic	p-value
Δ Average residue mass	3.56	0.47
Δ Charge	2.93	0.57
Δ Hydrophobicity	3.35	0.50
Δ Helix propensity	4.45	0.35
Δ Buried residues (%)	0.439	0.98
Δ Polarity ranking	2.35	0.67
Δ Volume (v/d Waals)	3.40	0.49

Table 7. Kruskal-wallis H stasitic and p-value for amino-acid substitutions

These results show that the hypothesis that the groups are sampled from the same distribution can not be rejected with an α any lower than 0.35. In addition, we ran separate tests comparing each group against the ‘unchanged’ group. In these 35 tests (5 groups, 7 properties) we found only one significant ($p < 0.05$) difference, in ‘Δ Helix propensity’ for ‘changed‘ versus ‘unchanged’, with $p = 0.026$. Given that this equates to a 1 in 38 chance of occurring randomly, and we performed 35 tests, we cannot attach any particular relevance to this finding.

9 Information gain per feature

The following five tables show the *information gain* of each feature in the machine-learning datasets, for the problems of predicting changes to activation, changes to inactivation, changes to the late component, and complete absence of I_{Na}, as well as the changed/unchanged outcome.

Gain	Feature	Gain	Feature
0.1048	Region type	0	Δ Volume (v/d Waals)
0.0959	Distance to any transmembrane segment	0	Δ Average residue mass
0.088	Segment type	0	Δ Charge
0.0814	Distance to segment 4 - segment 5 linker	0	Distance to C-terminus
0.0791	Conservedness score	0	Δ Helix-propensity
0.0777	Side	0	Distance to segment 5 - segment 6 linker
0.0771	Distance to segment 5	0	Distance to segment 6
0.0731	Distance to segment 4	0	Distance to linker domain III - domain IV
0	Δ Hydrophobicity	0	Substitution likelihood (Gonnet)
0	Δ % Buried residues	0	Amino-acid similarity (Grantham)
0	Δ Polarity ranking	0	Index on the gene

Table 8. Information gain: Activation

Gain	Feature	Gain	Feature
0.1829	Region type	0.0618	Distance to segment 4
0.1108	Distance to segment 5 - segment 6 linker	0	Δ Volume (v/d Waals)
0.0973	Distance to segment 5	0	Distance to segment 6
0.0927	Distance to linker domain III - domain IV	0	Δ Polarity ranking
0.0903	Conservedness score	0	Δ Average residue mass
0.0895	Distance to C-terminus	0	Δ % Buried residues
0.0895	Index on the gene	0	Δ Charge
0.0801	Segment type	0	Δ Hydrophobicity
0.079	Distance to any transmembrane segment	0	Substitution likelihood (Gonnet)
0.0697	Distance to segment 4 - segment 5 linker	0	Amino-acid similarity (Grantham)
0.0668	Side	0	Δ Helix-propensity

Table 9. Information gain: Inactivation

Gain	Feature	Gain	Feature
0.1154	Region type	0	Δ Helix-propensity
0.039	Side	0	Conservedness score
0.0357	Segment type	0	Amino-acid similarity (Grantham)
0	Distance to C-terminus	0	Distance to segment 5 - segment 6 linker
0	Substitution likelihood (Gonnet)	0	Distance to any transmembrane segment
0	Δ Hydrophobicity	0	Distance to segment 6
0	Δ Charge	0	Distance to segment 5
0	Δ Polarity ranking	0	Distance to linker domain III - domain IV
0	Δ Average residue mass	0	Distance to segment 4 - segment 5 linker
0	Δ % Buried residues	0	Distance to segment 4
0	Δ Volume (v/d Waals)	0	Index on the gene

Table 10. Information gain: Late component

Gain	Feature	Gain	Feature
0.2139	Region type	0	Distance to C-terminus
0.1356	Distance to segment 5 - segment 6 linker	0	Δ % Buried residues
0.1163	Side	0	Δ Hydrophobicity
0.1116	Distance to segment 4	0	Δ Charge
0.0999	Segment type	0	Conservedness score
0.0753	Distance to segment 4 - segment 5 linker	0	Δ Helix-propensity
0.0601	Distance to segment 5	0	Substitution likelihood (Gonnet)
0.049	Δ Average residue mass	0	Distance to segment 6
0.049	Δ Volume (v/d Waals)	0	Distance to linker domain III - domain IV
0.0458	Distance to any transmembrane segment	0	Amino-acid similarity (Grantham)
0	Δ Polarity ranking	0	Index on the gene

Table 11. Information gain: Zero current

Gain	Feature	Gain	Feature
0.1269	Region type	0	Δ Volume (v/d Waals)
0.1144	Distance to segment 5	0	Δ Charge
0.1119	Conservedness score	0	Δ Average Residue Mass
0.1073	Distance to segment 5 - segment 6 linker	0	Δ % Buried Residues
0.1019	Distance to any transmembrane segment	0	Δ Polarity ranking
0.0982	Distance to segment 4 - segment 5 linker	0	Distance to C-terminus
0.0836	Segment type	0	Δ Hydrophobicity
0.0821	Distance to segment 4	0	Amino-acid similarity (Grantham)
0.0754	Side	0	Substitution likelihood (Gonnet)
0.0521	Distance to segment 6	0	Δ Helix propensity
0.0488	Distance to linker domain III - domain IV	0	Index on the gene

Table 12. Information gain: Changed/unchanged

10 Tuning the classifiers

We investigated whether the performance of our classifiers could be improved by tuning them. For this, we defined parameter ranges for each classifier, and explored them exhaustively (i.e., a brute-force approach), in search of the best MCC. Tuning was performed on the training set (using 10-fold cross-validation to assess performance), and then later assessed on the independent test set (see main text). Details and results of tuning are given below.

Random forest For the random forest[14], we varied the 'break ties randomly' option, as well as 'bag size as percentage of the training set' (from 4 to 100 with step size 4), 'number of randomly chosen features' (from 0 to 10, step size 1), and 'number of iterations' (20 to 100, step size 10).

Naive Bayes The Naive Bayes classifier[15] offered only two parameters for tuning: a 'kernel estimator' switch, and a 'supervised discretisation' switch (which cannot be used at the same time). For this classifier we ran without, and with, both switches.

Multilayer perceptron For the multilayer perceptron classifier[16] (MLPClassifier), we varied the number of hidden units (2 to 10, with step size 1), the tolerance (10^{-1} to 10^{-9}, with a stepwise 10-fold decrease), and the 'ridge' (10^{-1} to 10^{-9}, with a stepwise 10-fold decrease). In all experiments we used an approximate sigmoid activation function and a squared-error loss function.

Support-vector machines The support vector machine classifier was created using LibSVM[17] using the default 'C-SVC' classification method and a radial basis function (RBF) kernel. We varied the misclassification penalty (from 2^0 to 2^{10} with a stepwise 2-fold increase), and the kernel bandwidth (from 2^{-25} to 2^{-5} with a stepwise 2-fold increase), and the kernel degree (from 1 to 10 with a stepwise increase of 1).

Nearest-neighbour For the nearest-neighbour classifier[18] (IBk), we varied the number of neighbours from 1 to 20. In addition, we tried 5 different distance functions (Chebyshev, Euclidean, Filtered distance, Manhattan, and Minkowski distance), and optional weighting (distance or 1/distance).

10.1 Results

A Naive Bayes classifier performed well for most problems. The multilayer perceptron (MLP) classifier could be made to fit well on the training/validation data set, but then faired poorly on the independent test set, indicating overfitting. The same phenomenon occurred to a lesser degree for the Random Forest classifiers. The simplest method, a nearest-neighbour classifier, performed worse than the other methods in most cases, but achieved the best result when predicting the presence of changes to the late component of I_{Na}, which proved to be the hardest problem. Supplemental Tables 13-17 show the performance of each classifier with and without tuning. The best results before and after tuning are highlighted in bold in each table.

Method	MCC	AUC	Acc.
Zero-R	0	0.5	62.9%
Random forest	**0.502**	0.687	77.4%
Random forest (tuned)	0.244	0.700	66.1%
Naive Bayes	0.325	0.697	66.1%
Naive Bayes (tuned)	**0.325**	0.657	66.1%
MLP	0.064	0.486	59.7%
MLP (tuned)	0.144	0.504	62.9%
SVM	0	0.5	62.9%
SVM (tuned)	-0.018	0.496	61.3%
Nearest-neighbour	0.097	0.546	59.7%
Nearest-neighbour (tuned)	0.214	0.655	64.5%

Table 13. Machine-learning results for activation

For activation, the Naive Bayes classifier without kernel estimation or supervised discretisation performed best. Performance of the Random Forest classifier, evaluated on the validation set, was worse after tuning it on the test set. This indicates that the method was exploiting structures found in one but not both sets. Since the sets were chosen at random, this indicates overfitting.

**Method**	**MCC**	**AUC**	**Acc.**
Zero-R	0	0.5	60.9%
Random forest	0.255	0.711	63.8%
Random forest (tuned)	0.287	0.755	66.7%
Naive Bayes	0.315	0.735	66.7%
Naive Bayes (tuned)	**0.305**	0.730	66.7%
MLP	**0.370**	0.744	71.0%
MLP (tuned)	0.208	0.727	63.8%
SVM	0	0.5	60.9%
SVM (tuned)	-0.072	0.483	58.0%
Nearest-neighbour	0.209	0.604	62.3%
Nearest-neighbour (tuned)	0.234	0.702	63.8%

Table 14. Machine-learning results for inactivation

For inactivation, the Naive Bayes classifier with kernel estimation had the best MCC. Notably, the MLP classifier performed worse on the test set after being tuned on the training data to have 5 hidden units, a tolerance of 10^{-7} and a ridge of 10^{-8}).

**Method**	**MCC**	**AUC**	**Acc.**
Zero-R	0	0.5	65.0%
Random forest	-0.019	0.455	57.5%
Random forest (tuned)	0.026	0.460	60.0%
Naive Bayes	**0.142**	0.291	57.5%
Naive Bayes (tuned)	-0.023	0.451	55.0%
MLP	-0.135	0.489	47.5%
MLP (tuned)	0.011	0.415	55.0%
SVM	-0.118	0.481	62.5%
SVM (tuned)	-0.010	0.497	62.5%
Nearest-neighbour	0.043	0.522	55.0%
Nearest-neighbour (tuned)	**0.061**	0.530	60.0%

Table 15. Machine-learning results for late current

All classifiers performed poorly on the late-I_{Na} problem. The best MCC was obtained using a *k*-nearest-neighbour method with a *k* of 13 and using a Chebyshev distance.

**Method**	**MCC**	**AUC**	**Acc.**
Zero-R	0	0.5	87.7%
Random forest	0.379	0.749	87.7%
Random forest (tuned)	0.345	0.798	86.4%
Naive Bayes	0.324	0.815	79.0%
Naive Bayes (tuned)	**0.584**	0.785	91.4%
MLP	**0.430**	0.744	87.7%
MLP (tuned)	0.449	0.737	86.4%
SVM	0	0.5	87.7%
SVM (tuned)	0.372	0.701	85.2%
Nearest-neighbour	0.372	0.701	85.2%
Nearest-neighbour (tuned)	0.379	0.799	87.7%

Table 16. Machine-learning results for zero current

The Naive Bayes classifier with kernel estimation performed best on the zero current problem.

Method	MCC	AUC	Acc.
Zero-R	0	0.5	72.8%
Random forest	0.120	0.656	66.7%
Random forest (tuned)	0.165	0.638	67.9%
Naive Bayes	0.221	0.659	65.4%
Naive Bayes (tuned)	**0.239**	0.693	66.7%
MLP	**0.257**	0.690	67.9%
MLP (tuned)	0.151	0.631	67.9%
SVM	0	0.5	72.8%
SVM (tuned)	0.120	0.557	66.7%
Nearest-neighbour	-0.028	0.557	66.7%
Nearest-neighbour (tuned)	0.058	0.576	67.9%

Table 17. Machine-learning results for changed/unchanged

For the changed/unchanged problem, we obtained the best results using a Naive Bayes classifier, this time with supervised discretisation enabled.

10.1.1 Discretised shifts in midpoint of inactivation

Method	AUC	Acc.
Zero-R	0.5	43.8%
Random forest	**0.706**	57.5%
Random forest (tuned)	**0.640**	46.6%
Naive Bayes	0.625	46.6%
Naive Bayes (tuned)	0.625	46.6%
MLP	0.610	41.1%
MLP (tuned)	0.606	49.3%
SVM	0.5	43.8%
SVM (tuned)	0.617	50.7%
Nearest-neighbour	0.648	50.7%
Nearest-neighbour (tuned)	0.626	45.2%

Table 18. Machine-learning results for discretised shifts in midpoint on inactivation

Midpoint of activation predictions were tuned by selecting the best AUC. For the changed/unchanged problem, we obtained the best results using a Random Forest classifier, as shown in Supplemental Table 18.

11 EP data overview

Supplemental Table 19 shows the EP data collected for this paper. The first columns show the EP-data reference and the variant. The next four indicate whether the authors considered the variant to affect activation, inactivation, or the late component, and whether the mutation abolished current completely. The next two columns show any reported shifts in midpoint of activation (ΔV_a) and inactivation (ΔV_i). Finally, the cell type, α-subunit, and co-expression of β_1-subunit are given. Cell types are HEK cells, CHO cells, Oocytes (Ooc.) or Mouse myocyte (MM). α-subunits are as defined in this paper (see supplementary Table 1), with the addition of a** which indicates the α-subunit used in a study by Chen et al.

Table 19. EP Data

Publication	Mutation	Act.	Inact.	Late	Zero	ΔV_a	ΔV_i	Cell	α	β_1
Guetter 2013[19]	G9V	no	no	no		1.3	0.01	HEK	a*	no
Guetter 2013[19]	R18Q	no	no			0.5	2.2	HEK	a*	no
Guetter 2013[19]	R18W	no	yes	no		-0.2	1.3	HEK	a*	no
Guetter 2013[19]	R27H	yes	yes			4	-0.4	HEK	a*	no
Kapplinger 2015[20]	E30G	no	yes	no		-0.2	2.6	HEK	b	no
Tan 2005[21]	R34C	no	no	no		-2	0	HEK	b	no
Tan 2005[21]	R34C	no	no	no		3	2	HEK	a	no
Guetter 2013[19]	G35S	no	no			-1.7	-1.8	HEK	a*	no
Lin 2008[22]	R43Q	no	no	no		-0.29	-1.77	HEK	a*	yes
Kapplinger 2015[20]	E48K	yes	no	no		3.9	-2.7	HEK	b	no
Kapplinger 2015[20]	E48K	no	no	no		3.8	0.4	HEK	a	no
Hoshi 2014[23]	N70K		no				-0.14	HEK	a	no
Kapplinger 2015[20]	Y87C	no	no	no		0.6	-1.5	HEK	b	no
Beyder 2014[24]	I94V	no	yes	no		-0.5	-1.3	HEK	b	no
Guetter 2013[19]	V95I	yes	no			1.2	1	HEK	a*	no
Clatot 2012[25]	R104K	yes	no			8.6	0	HEK		no
Guetter 2013[19]	R104Q	no	yes			-0.5	-1.6	HEK	a*	no
Clatot 2012[25]	R104W				yes			HEK		no
Clatot 2012[25]	R121W				yes			HEK		no
Holst 2010[26]	R121W				yes			HEK		no
Guetter 2013[19]	V125L	no	yes	no		0.7	3.1	HEK	a*	no
Guetter 2013[19]	K126E	no	yes			2.8	3.4	HEK	a*	no
Swan 2014[27]	I137V	no	no			-0.5	1.6	HEK	b	yes
Swan 2014[27]	I141V	yes	no			-7	1.5	HEK	b	yes
Gui 2010a[28]	E161K	yes	no			19.8	1.5	HEK	a*	no
Gui 2010b[29]	E161K	yes	no			19.1	1.9	HEK	a*	no
Smits 2005a[30]	E161K	yes	no			11.9	0.9	HEK	a*	no
Gui 2010a[28]	T187I				yes			HEK	a*	no
Gui 2010b[29]	T187I				yes			HEK	a*	no
Kapplinger 2015[20]	R190Q	no	no	no		0.1	0.8	HEK	b	no
Gui 2010a[28]	L212P	yes	yes			-15	-10.5	HEK	a*	no
Gui 2010b[29]	L212P	yes	yes			-14.6	-10.4	HEK	a*	no
Makita 2005[31]	L212P	yes	yes	no		-15.4	-9	HEK		yes
Kapplinger 2015[20]	S216L	no	no	no		-2	0.3	HEK	b	no
Marangoni 2011[32]	S216L	no	no	no		1	1	HEK	a*	yes
Wang 2007a[33]	S216L	no	yes	yes		-0.2	4.7	HEK	b	yes

Publication	Mutation	Act.	Inact.	Late	Zero	ΔV_a	ΔV_i	Cell	α	β_1
Abe 2014[34]	R219H	yes	yes			1.7	-11.4	HEK	b	no
Chen 1996[35]	R219H	yes	yes			-2.3	-9.6	Ooc.	a**	no
Gosselin-Badaroudine 2012[36]	R219H	no	no			-1.7	3.67	Ooc.		yes
Chen 1996[35]	R219Q	no	no			3.3	-0.8	Ooc.	a**	no
Beyder 2014[24]	T220I	no	yes	no		-2.1	-7.4	HEK	b	no
Gui 2010a[28]	T220I	yes	yes			-1	-5.6	HEK	a*	no
Gui 2010b[29]	T220I	no	yes			-1.8	-6.4	HEK	a*	no
Beckermann 2014[5]	R222Q	yes	yes	no		-13.9	-6.7	HEK		yes
Cheng 2010[1]	R222Q	yes	yes	no		-15.4	-8	HEK	a	no
Cheng 2010[1]	R222Q	yes	yes	no		-13.1	-4.2	HEK	b	no
Laurent 2012[2]	R222Q	yes	yes			-11.7	-4	COS	a	yes
Mann 2012[3]	R222Q	yes	yes			-6.3	-6.2	CHO		yes
Nair 2012[4]	R222Q	yes	yes	no		-9	-7.3	CHO	a	no
Chen 1996[35]	R225E				yes			Ooc.	a**	no
Beckermann 2014[5]	R225P	yes	yes	yes		0.2	-0.8	HEK		yes
Beckermann 2014[5]	R225Q	yes	yes	yes		-2.9	-4.2	HEK		yes
Chen 1996[35]	R225Q	yes	yes			4.7	5.8	Ooc.	a**	no
Bezzina 2003[37]	R225W	yes	yes	no		14	11.1	Ooc.	a*	yes
Hoshi 2014[23]	R225W		no				-1.81	HEK	a	no
Watanabe 2011a[38]	A226D				yes			HEK		yes
Neu 2010[39]	I230T	yes	yes			15.4	-4.9	HEK	a*	yes
Kapplinger 2015[20]	Q245K	no	no	no		-3.7	0.7	HEK	b	no
Calloe 2011[40]	Q270K	yes	yes	yes		5.8	9.9	CHO	b	yes
Itoh 2005a[41]	R282H				yes	5	5	HEK	a*	yes
Poelzing 2006[42]	R282H				yes			HEK	a	no
Shinlapawittayatorn 2011a[43]	R282H				yes			HEK	a	no
Shinlapawittayatorn 2011a[43]	G292S		yes				7.3	HEK	a	no
Shinlapawittayatorn 2011a[43]	V294M						4.1	HEK	a	no
Saito 2009[44]	G298S	no	no					HEK	a	no
Saito 2009[44]	G298S	no	no					HEK	b	no
Wang 2002[45]	G298S	no	yes	no		3.6	7.4	HEK	a*	yes
Shinlapawittayatorn 2011a[43]	K317N				yes			HEK	a	no
Keller 2005[46]	L325R	yes	yes			10.4	4.4	HEK	b*	yes
Shinlapawittayatorn 2011a[43]	L325R				yes			HEK	a	no
Cordeiro 2006[47]	P336L	no	no			1.7	-1.5	HEK		yes
Olesen 2012[48]	R340Q	yes	yes	no		-6.2	-5.5	HEK		no
Shinlapawittayatorn 2011a[43]	G351V				yes			HEK	a	no
Vatta 2002a[49]	G351V	no	no			0	2.1	Ooc.		no
Guo 2016[50]	Y352C	no	yes			0		HEK	a	no
Pfahnl 2007[51]	T353I	no	yes	yes		1	11	HEK	b*	no
Zhang 2015[52]	T353I	yes	yes	yes		-2.2	-6.32	HEK		no
Shinlapawittayatorn 2011a[43]	D356N				yes			HEK	a	no
Hong 2004[53]	R367H				yes			HEK	a*	no
Shinlapawittayatorn 2011a[43]	R367H				yes			HEK	a	no

Publication	Mutation	Act.	Inact.	Late	Zero	ΔV_a	ΔV_i	Cell	α	β_1
Vatta 2002b[54]	R367H				yes			Ooc.	b	no
Watanabe 2011a[38]	R367H				yes			HEK		yes
Detta 2014[55]	R376C	yes	no			11.5	0	HEK	a*	yes
Detta 2014[55]	R376H	no	no			1.5	0	HEK	a*	yes
Frustaci 2005[56]	R376H				yes			HEK		yes
Rossenbacker 2004[57]	R376H	no	no			0	0	HEK		yes
Kapplinger 2015[20]	I397F	yes	yes	yes		-6.9	5.1	HEK	b	no
Kapplinger 2015[20]	I397F	no	yes	yes		0.4	9.1	HEK	a	no
Hu 2007[58]	G400A	no	yes	no		1.32	-6.39	HEK	b*	yes
Kato 2014[59]	N406K	yes	yes	yes		8.6	2.9	CHO	a*	yes
Itoh 2005b[60]	N406S	yes	yes			15.9	9.6	HEK	a*	yes
Itoh 2007[61]	N406S		yes	no			2.7	HEK	a*	yes
Horne 2011[62]	V411M	yes	yes	yes		-8.1	-7.9	HEK		no
Hoshi 2014[23]	E439K		no				-0.52	HEK	a	no
Crotti 2012a[63]	E446K	no	yes			-0.7	-6.2	HEK		yes
Kapplinger 2015[20]	E462A	no	no	no		-2.6	1.2	HEK	b	no
Kapplinger 2015[20]	E462A	no	no	no		-2.2	0.9	HEK	a	no
Kapplinger 2015[20]	E462K	no	no	no		-1.9	-3	HEK	b	no
Holst 2009[64]	P468L	no	no			-1.4	2.8	HEK		no
Tan 2005[21]	R481W	no	yes	no		1	-6	HEK	b	no
Tan 2005[21]	R481W	no	yes	no		-6	-4	HEK	a	no
Viswanathan 2003[65]	T512I	yes	yes			-7.5	-8.7	HEK	a*	yes
Tan 2001[66]	G514C	yes	yes	no		10.1	6.9	HEK		no
Shuraih 2007[67]	S524Y	no	no			-5	1.6	HEK	b	no
Tan 2005[21]	S524Y	no	no	no		-1	-2	HEK	b	no
Tan 2005[21]	S524Y	no	no	no		3	3	HEK	a	no
Aiba 2014[68]	R526H	no	no			-0.2	-0.5	HEK		yes
Hoshi 2014[23]	R526H		no				-1.81	HEK	a	no
Aiba 2014[68]	S528A	no	no			-2.2	0.4	HEK		yes
Otagiri 2008[69]	F532C	no	no	no		0	0	HEK		yes
Chiang 2009[70]	A551E		no				0	HEK		yes
Chiang 2009[70]	A551T	no	yes			0	-5	HEK		yes
Juang 2014a[71]	A551T	yes	no			-1.8	-1.4	HEK	a	yes
Chiang 2009[70]	A551V		yes				-4.7	HEK		yes
Hoshi 2014[23]	G552R		no				-1.75	HEK	a	no
Hoshi 2014[23]	E555K		no				-2.68	HEK	a	no
Cheng 2010[1]	H558R	no	no	no		-0.9	-1	HEK	a	no
Cheng 2010[1]	H558R	no	no	no		-0.3	0.6	HEK	b	no
Gui 2010b[29]	H558R	no	no			-1.7	-1.5	HEK	a*	no
Kauferstein 2013a[72]	H558R	no	no			2.55	-1	Ooc.		no
Murphy 2012[73]	H558R	no	no			5.1	-0.8	HEK	a*	no
Surber 2008[74]	H558R	no	no	no		0.6	-2.2	Ooc.	a*	no
Tan 2005[21]	H558R	no	no	no		0	-6	HEK	b	no
Tan 2005[21]	H558R				yes			HEK	a	no
Hoshi 2014[23]	L567Q		no				-0.43	HEK	a	no

Publication	Mutation	Act.	Inact.	Late	Zero	ΔV_a	ΔV_i	Cell	α	β_1
Wan 2001[75]	L567Q	yes	yes			7.1	-11.3	HEK		yes
Kapplinger 2015[20]	R569G	yes	no	no		-7.1	0.7	HEK	b	no
Kapplinger 2015[20]	R569W	no	no	no		-1.3	0.7	HEK	b	no
Glynn 2015[76]	S571A		no	yes			0	MM		yes
Glynn 2015[76]	S571E		no	yes			0	MM		yes
Albert 2008[77]	A572D	no	yes			-2.5	0.8	Ooc.	a*	yes
Tester 2010[78]	A572D	no	no	no		0	-3	HEK	b	no
Albert 2008[77]	A572F	no	yes			-3.8	-2.4	Ooc.	a*	yes
Juang 2014a[71]	N592K	yes	no			3.2	1.2	HEK	a	yes
Albert 2008[77]	G615E	no	yes			-3.4	1.7			yes
Beyder 2014[24]	G615E	yes	yes	no		5.6	3.1	HEK	b	no
Yang 2002[79]	G615E	no	no	no		0.5	2.4	HEK	a*	yes
Yang 2002[79]	L618F	no	no	no		-4.9	3.9	HEK	a*	yes
Wehrens 2003[80]	L619F	no	yes	yes		-0.1	5.8	HEK		yes
Hoshi 2014[23]	R620C		no				-2.17	HEK	a	no
Kapplinger 2015[20]	R620C	no	yes	no		0.5	2.3	HEK	b	no
Calloe 2013[81]	R620H	no	no			0	0	CHO	b	no
Kapplinger 2015[20]	P627L	no	no	no		-1.1	1	HEK	b	no
Beyder 2014[24]	T630M	no	yes	no		1.5	1.3	HEK	b	no
Hoshi 2014[23]	T632M		no				-2.33	HEK	a	no
Hoshi 2014[23]	A647D		no				-1.06	HEK	a	no
Beyder 2014[24]	P648L	no	yes	no		2	-0.5	HEK	b	no
Cheng 2011[82]	R680H	no	no	yes		1.1	2.4	HEK	b	no
Cheng 2011[82]	R680H	no	no	no		-2.6	-1.5	HEK	a	no
Wang 2007a[33]	R680H	no	yes	no		-1.9	0.1	HEK	b	yes
Mok 2003[83]	H681P	yes	yes			-9.5	-17.3	HEK		yes
Sottas 2013[84]	R689C	no	no	yes		0.1	-0.2	HEK		yes
Hong 2012[85]	R689H				yes					no
Kapplinger 2015[20]	R689H	yes	yes	no		-5.2	-4.3	HEK	b	no
Sottas 2013[84]	R689H	no	no	yes		-0.7	-0.6	HEK		yes
Kapplinger 2015[20]	Q692K	yes	no	no		-2.2	-4.3	HEK	b	no
Kapplinger 2015[20]	Q692K	no	no	no		-1.1	2.3	HEK	a	no
Hoshi 2014[23]	P701L		no				-3.04	HEK	a	no
Vatta 2002b[54]	A735V	yes	yes			6.7	-0.1	Ooc.	b	no
Kapplinger 2015[20]	Q750R	no	no	no		3.2	-1.6	HEK	b	no
Kapplinger 2015[20]	Q750R	no	yes	no		0.3	-2.5	HEK	a	no
Potet 2003[86]	G752R				yes			COS		no
Kapplinger 2015[20]	R800L	no	no	no		-1	0.9	HEK	b	no
Chen 1996[35]	R808H	yes	yes			6.3	0	Ooc.	a**	no
Chen 1996[35]	R808Q	yes	yes			13.3	8.2	Ooc.	a**	no
Calloe 2013[81]	R811H	no	yes			-0.6	-8.4	CHO	b	no
Wang 2015[87]	L812Q	no	yes			-0.33	-19.7	HEK	a*	no
Chen 1996[35]	R814E	yes	no			15.8	-2.4	Ooc.	a**	no
Chen 1996[35]	R814Q	yes	yes			0.4	-9.6	Ooc.	a**	no
Beckermann 2014[5]	R814W	yes	yes	no		-3.8	1.1	HEK		yes

Publication	Mutation	Act.	Inact.	Late	Zero	ΔV_a	ΔV_i	Cell	α	β_1
Nguyen 2008[88]	R814W	yes	yes			-5.7	-2.9	HEK	a*	yes
Kinoshita 2016[89]	K817E	yes	yes			24	0.1	HEK	a	yes
David 2012[90]	L828F	yes	no			-21.7	-8.4			no
Watanabe 2011a[38]	L846R				yes			HEK		yes
Clatot 2012[25]	R878C				yes			HEK		no
Gui 2010a[28]	R878C				yes			HEK	a*	no
Gui 2010b[29]	R878C				yes			HEK	a*	no
Zhang 2008[91]	R878C				yes			HEK	a*	yes
Zhang 2008[91]	R878C				yes			HEK	a*	no
Zhang 2008[91]	R878C				yes			Ooc.	a*	no
Zhang 2008[91]	R878K				yes			HEK	a*	no
Tarradas 2013[92]	I890T	yes	no	no		4.7	0.7	HEK	a*	no
Kapplinger 2015[20]	G897E				yes			HEK	b	no
Wang 2007b[93]	N927K	yes	yes			-7.1		HEK	a*	yes
Ruan 2007[94]	S941N	no	no	yes		-0.6	-0.3	HEK		yes
Schwartz 2000[95]	S941N			yes				Ooc.	b*	no
Hsueh 2009[96]	R965C	no	yes			1.45	-9.4	HEK	a	yes
Hoshi 2014[23]	R965H		no				-0.3	HEK	a	no
Hayashi 2015[97]	R986Q	no	no			1.3	-0.2	CHO		yes
Beyder 2014[24]	A997T	yes	yes	no		19.6	6.2	HEK	b	no
Hu 2010[98]	P1008S	no	no				1.37	HEK		yes
Frustaci 2005[56]	R1023H	no	yes			3.4	2.5	HEK		yes
Hoshi 2014[23]	R1023H		no				2.25	HEK	a	no
Hoshi 2014[23]	E1053K		no				3.71	HEK	a	no
Mohler 2004[99]	E1053K	yes	yes			-8.3	-4.7	HEK	a*	yes
Otagiri 2008[69]	G1084S	yes	yes	no		1.5	-5.5	HEK		yes
Juang 2014a[71]	P1090L	no	no			-0.5	-1.5	HEK	a	yes
Tan 2005[21]	P1090L	yes	no	no		-5	-4	HEK	b	no
Tan 2005[21]	P1090L	no	no	no		1	3	HEK	a	no
Cheng 2011[82]	S1103Y	yes	no	no		1.8	6.3	HEK	b	no
Cheng 2011[82]	S1103Y	no	yes	no		-1.6	-2.6	HEK	a	no
Splawski 2002[100]	S1103Y	yes	no	yes		-4.5	0	HEK	b	no
Tan 2005[21]	S1103Y	yes	yes	no		-3	-3	HEK	b	no
Tan 2005[21]	S1103Y	yes	yes	no		5	6	HEK	a	no
Hoshi 2014[23]	A1113V		no				-0.78	HEK	a	no
Hoshi 2014[23]	S1140T		no				-3.05	HEK	a	no
Beyder 2014[24]	G1158S	no	yes	no		3.2	-3.3	HEK	b	no
Winkel 2012[101]	P1177L	no	no	yes		0.2	-1.5	HEK		no
Ge 2008[102]	A1180V	no	yes	yes		1.4	-4.4	HEK	a	yes
Huang 2006[103]	R1193Q	no	yes	yes		2	-5.2	HEK		no
Tan 2005[21]	R1193Q	no	no	no		-1	-2	HEK	b	no
Tan 2005[21]	R1193Q	yes	yes	no		-6	-5	HEK	a	no
Vatta 2002b[54]	R1193Q	no	yes			0	3.9	Ooc.	b	no
Wang 2004[104]	R1193Q	no	yes	yes		0	-6	HEK	a	no
Medeiros-Domingo 2009[105]	R1195H	yes	yes	no		-9.2	-10	HEK	b	no

Publication	Mutation	Act.	Inact.	Late	Zero	ΔV_a	ΔV_i	Cell	α	β_1
Albert 2008[77]	W1206C	no	no			-2.6	0.8	Ooc.	a*	yes
Calloe 2013[81]	S1218I				yes			CHO	b	no
Yang 2002[79]	F1250L	no	no	no		7.5	6.2	HEK	a*	yes
Groenewegen 2003a[106]	D1275N	no	no			3.36	-0.7	Ooc.	a*	yes
Groenewegen 2003a[106]	D1275N	yes	yes			3.8	-4.05	Ooc.	a*	no
Gui 2010a[28]	D1275N	yes	yes			3.1	1.8	HEK	a*	no
Gui 2010b[29]	D1275N	yes	yes			3	1	HEK	a*	no
Hoshi 2014[23]	D1275N		no				-0.84	HEK	a	no
Watanabe 2011b[107]	D1275N	no	no			0.7	-3.9	CHO	a	no
Watanabe 2011b[107]	D1275N	yes	no			12	1.4	HEK	a	yes
Watanabe 2011b[107]	D1275N	no	yes	yes		-1.5	7.6	MM		no
Abriel 2001[108]	E1295K	yes	yes			2.9	5.2	HEK	a*	yes
Liu 2002[10]	E1295K	yes	yes			3.4	5.2	HEK	a*	yes
Gui 2010a[28]	P1298L	no	yes			-0.5	-8.9	HEK	a*	no
Gui 2010b[29]	P1298L	no	yes			-1.4	-10.1	HEK	a*	no
Chen 1996[35]	K1300H	yes	yes			0.3	-13.1	Ooc.	a**	no
Chen 1996[35]	K1300Q	yes	yes			-10.4	-21	Ooc.	a**	no
Beyder 2014[24]	T1304M	no	no	no		-0.3	0.6	HEK	b	no
Kapplinger 2015[20]	T1304M	no	no	no		-3.5	-0.7	HEK	b	no
Wang 2007a[33]	T1304M	yes	yes	yes		6.7	11.2	HEK	b	yes
Chen 1996[35]	R1306E	yes	yes			-2.1	-21.8	Ooc.	a**	no
Chen 1996[35]	R1306Q	yes	yes			5.9	-10.6	Ooc.	a**	no
Wang 2016[109]	R1309H	yes	yes	yes		4.5	-6.2	HEK		yes
Casini 2007[110]	G1319V	yes	yes	no		3.7	-6	HEK		yes
Hoshi 2014[23]	G1319V		no				-1.92	HEK	a	no
Kapplinger 2015[20]	M1320V	no	no	no		0.7	2.4	HEK	b	no
Wang 1996[111]	N1325S	yes	yes	yes		-6.4	-2.5	HEK	a*	no
Yong 2007[112]	N1325S	no	yes	yes		0	7.1			no
Wedekind 2001[113]	A1330P	no	yes	no		-1.5	8.3	HEK	a*	yes
Smits 2005b[114]	A1330T	no	yes	no		2.4	6.9	HEK	a*	yes
Ruan 2007[94]	P1332L	yes	yes	no		-5	-6.4	HEK		yes
Huang 2009[115]	S1333Y	yes	yes	yes		-8.5	7	HEK	a*	yes
Samani 2009[116]	V1340I	no	no			-1.1	3.6	HEK	b	yes
Samani 2009[116]	V1340I				yes			HEK	a	yes
Keller 2006[117]	F1344S	yes	yes			9.7	-0.65	HEK		yes
Kyndt 2001[118]	G1406R				yes			Ooc.	a*	no
Kyndt 2001[118]	G1406R				yes			Ooc.	a*	yes
Tan 2006[119]	G1406R	no	yes	no		1.9	-10.5	HEK	a	no
Tan 2006[119]	G1406R	no	yes	no		3.4	-6.3	HEK	b	no
Gui 2010a[28]	G1408R				yes			HEK	a*	no
Gui 2010b[29]	G1408R				yes			HEK	a*	no
Baroudi 2001[120]	R1432C				yes			HEK	a*	yes
Baroudi 2001[120]	R1432G				yes			HEK	a*	yes
Baroudi 2001[120]	R1432G	no	no			0	0	Ooc.	a*	yes
Deschenes 2000[121]	R1432G				yes			HEK	a*	no

Publication	Mutation	Act.	Inact.	Late	Zero	ΔV_a	ΔV_i	Cell	α	β_1
Baroudi 2001[120]	R1432H				yes			HEK	a*	yes
Baroudi 2001[120]	R1432K	no	no			-4.94	0.2	HEK	a*	yes
Six 2008[122]	P1438L				yes			HEK		yes
Sarhan 2009[123]	F1473A		yes				14.8	HEK	a	no
Bankston 2007b[124]	F1473C	no	yes	yes		-2.4	8.8	HEK	a*	yes
Ruan 2010[125]	F1473S	yes	yes	yes		-18.9	-4.4	HEK		yes
Moreau 2013[126]	Q1476R	no	yes	yes		1.6	6.5	HEK	a*	yes
Kapplinger 2015[20]	I1485V	no	yes	no		-2.5	6.8	HEK	b	no
Wang 2007a[33]	F1486L	yes	yes	yes		3.7	14.3	HEK	b	yes
Li 2009[127]	K1493R	no	yes	no		-1.58	5.13	HEK		no
Sarhan 2009[123]	Y1494A		yes				7.9	HEK	a	no
Sarhan 2009[123]	Y1495A		no				2.6	HEK	a	no
Hoshi 2014[23]	L1501V		no				0.19	HEK	a	no
Hoshi 2014[23]	G1502S		yes				-7.18	HEK	a	no
Saber 2015[128]	P1506S	yes	yes			9	-14	HEK		yes
Beyder 2014[24]	R1512Q	no	no	no		2.9	3	HEK	b	no
Deschenes 2000[121]	R1512W	no	yes			0.9	2.6	HEK	a*	no
Rook 1999[129]	R1512W	yes	yes			-5.1	-3.8	Ooc.	a*	no
Sarhan 2009[123]	F1520A		no				1.1	HEK	a	no
Sarhan 2009[123]	F1522A		no				-0.6	HEK	a	no
Nguyen 2008[88]	D1595H	no	yes			-0.3	-6.8	HEK	a*	yes
Wang 2002[45]	D1595N	no	yes	no		-1.3	4.2	HEK	a*	yes
Surber 2008[74]	T1620H	no	yes			-0.03	1.26	Ooc.	a*	no
Surber 2008[74]	T1620K	yes	yes	yes		-5.8	-4.14	Ooc.	a*	no
Baroudi 2000a[130]	T1620M	no	yes	no		0	0	HEK	a*	yes
Baroudi 2000a[130]	T1620M	no	yes	no		0	9.6	Ooc.	a*	yes
Shirai 2002[131]	T1620M	yes	yes			6.8	13.1	HEK	a*	no
Wang 2000[132]	T1620M	no	yes			0	6.2	HEK	a*	yes
Surber 2008[74]	T1620R	yes	yes			-6.56	-13.5	Ooc.	a*	no
Chen 1996[35]	R1623H	yes	yes			-2.4	-0.9	Ooc.	a**	no
Chen 1996[35]	R1623Q	yes	yes			2.3	4.7	Ooc.	a**	no
Kambouris 2000[133]	R1623Q		yes			0	-7.2	Ooc.		yes
Makita 1998[134]	R1623Q	no	yes	yes			-1.4	Ooc.	a*	no
Tsurugi 2009[135]	R1623Q	no	no	no		-0.9	-3.8	HEK		no
Olesen 2012[48]	R1626H	yes	yes	yes		4.4	-5.6			no
Ruan 2007[94]	R1626P	no	yes	yes		-3.1	-7.1	HEK		yes
Chen 1996[35]	R1629E	yes	yes			6.7	-3.7	Ooc.	a**	no
Chen 1996[35]	R1629Q	yes	yes			8.6	-57.5	Ooc.	a**	no
Zeng 2013[136]	R1629Q	no	yes			2.3	-20.6	HEK		yes
Wang 2008[137]	G1631D	yes	yes	yes		7.8	14.5	HEK	a*	yes
Nakajima 2015[138]	R1632C	no	yes	no		-2.2	-24.8	HEK	b	yes
Gui 2010a[28]	R1632H	no	yes			-1.4	-20.7	HEK	a*	no
Gui 2010b[29]	R1632H	no	yes			-2.4	-22	HEK	a*	no
Frustaci 2005[56]	R1644C	yes	yes			8.48	-1	HEK		yes
Wang 1996[111]	R1644H	no	yes	yes		1.7	2.1	HEK	a*	no

Publication	Mutation	Act.	Inact.	Late	Zero	ΔV_a	ΔV_i	Cell	α	β_1
Ruan 2007[94]	M1652R	no	yes	yes		0.7	7.6	HEK		yes
Cordeiro 2006[47]	I1660V				yes			HEK		yes
Nunez 2013[139]	D1690N	no	no	no		4.7	3.2	CHO	a*	yes
Otagiri 2008[69]	F1705S	yes	yes	no		-1	-17	HEK		yes
Akai 2000[140]	S1710L	yes	yes			17.7	-24.3	HEK	a*	yes
Shirai 2002[131]	S1710L	yes	yes			18.7	-21.7	HEK	a*	no
Amin 2005[141]	D1714G	no	yes			1.9	1.9	HEK		yes
Baroudi 2004[142]	G1740R				yes			HEK		no
Valdivia 2004[143]	G1743R				yes			HEK	b	no
Nunez 2013[139]	G1748D	yes	yes	no		13.4	13.8	CHO	a*	yes
Chang 2004[144]	I1762A	no	yes	no		4.1	11.8	HEK		yes
Chang 2004[144]	V1763M	yes	yes	yes		-3.8	11.5	HEK		yes
Chang 2004[144]	V1764M	no	yes	yes		-0.7	6	HEK		yes
Valdivia 2002[145]	M1766L	no	yes	yes		7	9	HEK	b*	yes
Groenewegen 2003b[146]	I1768V	no	yes	no		0.7	0.9	Ooc.	a*	no
Kauferstein 2013a[72]	I1768V	yes	yes			-6.7	-1.1	Ooc.		no
Rivolta 2002[147]	I1768V	no	yes	no		0	7.6	HEK		yes
Kato 2014[59]	N1774D	yes	yes	yes		-7.9	-0.9	CHO	a*	yes
Lupoglazoff 2001[148]	V1777M	yes	yes	yes		-8.99	-12.4	HEK		yes
Kapplinger 2015[20]	T1779M	no	yes	no		-3.1	-3.6	HEK	b	no
Beyder 2014[24]	E1780G	no	yes	no		3.6	2.1	HEK	b	no
Deschenes 2000[121]	E1784K	yes	yes	yes		8.8	-14.4	HEK	a*	no
Hu 2014[149]	E1784K	no	yes	yes		0	-18.4	HEK	b	no
Makita 2008[150]	E1784K	yes	yes	yes		12.5	-15	HEK		yes
Wei 1999[151]	E1784K		yes	yes			-12.1	Ooc.	a*	yes
Hu 2015[152]	S1787N	no	no	yes		-1	1	HEK	b	no
Hu 2015[152]	S1787N	no	no	no		-1	-1	HEK	a	no
Abriel 2000[7]	D1790G		yes				-15.6	HEK	a*	yes
An 1998[6]	D1790G	no	no	no		0	-2.9	HEK	a*	no
An 1998[6]	D1790G	no	yes	no		0	-16.3	HEK	a*	yes
Baroudi 2000b[8]	D1790G	yes	yes	yes		5.36	-14.6	HEK	a*	yes
Liu 2002[10]	D1790G	no	yes			1.4	-10.2	HEK	a*	yes
Liu 2003a[11]	D1790G	yes	yes			8.49	-22.3	HEK		no
Wehrens 2000[9]	D1790G	yes	yes	no		6	-15	HEK	a*	no
Liu 2002[10]	Y1795C	no	yes			-1	-2.8	HEK	a*	yes
Rivolta 2001[153]	Y1795C	no	yes	yes		-1	-10.5	HEK		yes
Tateyama 2003[154]	Y1795C		yes	yes		0	-11	HEK		yes
Liu 2002[10]	Y1795E	no	yes			1.7	-10.4	HEK	a*	yes
Liu 2002[10]	Y1795H	no	yes			0.9	-10.5	HEK	a*	yes
Rivolta 2001[153]	Y1795H	no	yes	yes		1.1	-10.9	HEK		yes
Tateyama 2003[154]	Y1795H		yes	yes		0	-11	HEK		yes
Liu 2002[10]	Y1795R	no	yes			2.1	-10.6	HEK	a*	yes
Kapplinger 2015[20]	D1819N	no	no	no		-1.9	-0.9	HEK	b	no
Olesen 2012[48]	D1819N	no	yes	yes		0.1	0.2	HEK		no
Liu 2005[155]	L1825P	no	yes	yes		0	-7.3	CHO	b	no

Publication	Mutation	Act.	Inact.	Late	Zero	ΔV_a	ΔV_i	Cell	α	β_1
Makita 2002[156]	L1825P	yes	yes	yes		8.9	-11	HEK	a*	yes
Cheng 2010[1]	I1836T	no	no	no		0.5	0.5	HEK	a	no
Cheng 2010[1]	I1836T	no	no	no		0.4	1.7	HEK	b	no
Musa 2015[157]	H1849R	no	yes			-0.5	-6.7	HEK		no
Petitprez 2008[158]	C1850S	no	yes			1.4	-11.6	HEK	b*	yes
Beyder 2014[24]	A1870D	no	yes	no		0.6	-1.1	HEK	b	no
Makiyama 2008[159]	M1875T	no	yes	no		-0.48	16.4	HEK	a*	yes
Beyder 2014[24]	L1896V	no	no	no		0.5	-2.6	HEK	b	no
Olesen 2012[48]	R1897W	no	yes	no		-1.6	-6.2	HEK		no
Bankston 2007a[160]	S1904L	no	yes	yes		-1.3	-4.9	HEK		yes
Glaaser 2012[161]	S1904L		no	yes			0.6	HEK		no
Rook 1999[129]	A1924T	yes	no			-9	-0.2	Ooc.	a*	no
Tan 2002[162]	A1924T	no	yes					HEK	a*	no
Hoshi 2014[23]	E1938K		no				-0.96	HEK	a	no
Shinlapawittayatorn 2011b[163]	V1951L		yes	no			8.8	HEK	a	no
Tan 2005[21]	V1951L	no	no	no		-1	3	HEK	b	no
Tan 2005[21]	V1951L	no	no	no		0	-2	HEK	a	no
Wang 2007a[33]	V1951L	no	yes	no		-1.6	1.8	HEK	b	yes
Olesen 2012[48]	V1951M	no	no	no		-3.6	0	HEK		no
Beyder 2014[24]	M1952T	no	no	no		0.2	4	HEK	b	no
Frustaci 2005[56]	I1968S	no	yes			0	-0.3	HEK		yes
Ellinor 2008[164]	N1987K	no	yes	no		-1.3	-3.4	Ooc.	a*	yes
Bebarova 2008[165]	F2004L	yes	yes	yes		3.3	-7.5	CHO	a	no
Wang 2007a[33]	F2004L	no	yes	yes		0.7	4.7	HEK	b	yes
Shinlapawittayatorn 2011b[163]	P2006A		yes	yes			10.6	HEK	a	no
Wang 2007a[33]	P2006A	no	yes	yes		-0.2	4.7	HEK	b	yes
Chen 2016[166]	V2016M	yes	no	no		0.8	-1.8	HEK		yes
Shy 2014[167]	V2016M	yes	no			3.7	1.9	HEK		no

References

1. Cheng, J. *et al.* SCN5A rare variants in familial dilated cardiomyopathy decrease peak sodium current depending on the common polymorphism H558R and common splice variant Q1077del. *Clin. Transl. Sci.* **3**, 287–294 (2010).

2. Laurent, G. *et al.* Multifocal ectopic Purkinje-related premature contractions: A new SCN5A-related cardiac channelopathy. *J. Am. Coll. Cardiol.* **60**, 144–156 (2012).

3. Mann, S. A. *et al.* R222Q SCN5A mutation is associated with reversible ventricular ectopy and dilated cardiomyopathy. *J. Am. Coll. Cardiol.* **60**, 1566–1573 (2012).

4. Nair, K. *et al.* Escape capture bigeminy: phenotypic marker of cardiac sodium channel voltage sensor mutation R222Q. *Heart Rhythm.* **9**, 1681–1688 (2012).

5. Beckermann, T. M., McLeod, K., Murday, V., Potet, F. & George, A. L. Novel SCN5A mutation in amiodarone-responsive multifocal ventricular ectopy-associated cardiomyopathy. *Heart Rhythm.* **11**, 1446–1453 (2014).

6. An, R. *et al.* Novel LQT-3 mutation affects Na+ channel activity through interactions between α-and β1-subunits. *Circ. Res.* **83**, 141–146 (1998).

7. Abriel, H., Wehrens, X., Benhorin, J., Kerem, B. & Kass, R. Molecular pharmacology of the sodium channel mutation D1790G linked to the long-QT syndrome. *Circulation* **102**, 921–925 (2000).

8. Baroudi, G. & Chahine, M. Biophysical phenotypes of SCN5A mutations causing long QT and Brugada syndromes. *FEBS Lett.* **487**, 224–228 (2000).

9. Wehrens, X., Abriel, H., Cabo, C., Benhorin, J. & Kass, R. Arrhythmogenic mechanism of an LQT-3 mutation of the human heart Na+ channel α-subunit: A computational analysis. *Circulation* **102**, 584–590 (2000).

10. Liu, H., Tateyama, M., Clancy, C. E., Abriel, H. & Kass, R. S. Channel openings are necessary but not sufficient for use-dependent block of cardiac Na+ channels by flecainide evidence from the analysis of disease-linked mutations. *J. Gen. Physiol.* **120**, 39–51 (2002).

11. Liu, C.-j., Dib-Hajj, S. D., Renganathan, M., Cummins, T. R. & Waxman, S. G. Modulation of the cardiac sodium channel NaV1.5 by fibroblast growth factor homologous factor 1B. *J. Biol. Chem.* **278**, 1029–1036 (2003).

12. Krawczak, M., Ball, E. V. & Cooper, D. N. Neighboring-nucleotide effects on the rates of germ-line single-base-pair substitution in human genes. *The Am. J. Hum. Genet.* **63**, 474–488 (1998).

13. Zhao, Z. & Boerwinkle, E. Neighboring-nucleotide effects on single nucleotide polymorphisms: a study of 2.6 million polymorphisms across the human genome. *Genome research* **12**, 1679–1686 (2002).

14. Breiman, L. Random forests. *Mach. Learn.* **45**, 5–32 (2001).

15. John, G. H. & Langley, P. Estimating continuous distributions in bayesian classifiers. In *Proceedings of the Eleventh conference on Uncertainty in artificial intelligence*, 338–345 (Morgan Kaufmann Publishers Inc, 1995).

16. Bouckaert, R. R. *et al.* *WEKA Manual for Version 3-9-1* (The University of Waikato, 2016).

17. Chang, C.-C. & Lin, C.-J. LIBSVM: a library for support vector machines. *ACM T. on Intell. Syst. Technol.* **2**, 27 (2011).

18. Aha, D. W., Kibler, D. & Albert, M. K. Instance-based learning algorithms. *Mach. Learn.* **6**, 37–66 (1991).

19. Gütter, C., Benndorf, K. & Zimmer, T. Characterization of N-terminally mutated cardiac Na+ channels associated with long QT syndrome 3 and Brugada syndrome. *Front. Physiol.* 41 (2013).

20. Kapplinger, J. *et al.* Enhanced classification of Brugada syndrome-associated and long-QT syndrome-associated genetic variants in the SCN5A-encoded Na(v)1.5 cardiac sodium channel. *Circ. Cardiovasc. Genet.* **8**, 582–595 (2015).

21. Tan, B.-H. *et al.* Common human SCN5A polymorphisms have altered electrophysiology when expressed in PQ1077 splice variants. *Heart Rhythm.* **2**, 741–747 (2005).

22. Lin, M.-T. *et al.* In utero onset of long QT syndrome with atrioventricular block and spontaneous or lidocaine-induced ventricular tachycardia: compound effects of hERG pore region mutation and SCN5A N-terminus variant. *Heart Rhythm.* **5**, 1567–1574 (2008).

23. Hoshi, M. *et al.* Brugada syndrome disease phenotype explained in apparently benign sodium channel mutations. *Circ. Cardiovasc. Genet.* **7**, 123 (2014).

24. Beyder, A. *et al.* Loss-of-function of the voltage-gated sodium channel NaV1.5 (channelopathies) in patients with irritable bowel syndrome. *Gastroenterol.* **146**, 1659–1668 (2014).

25. Clatot, J. *et al.* Dominant-negative effect of SCN5A N-terminal mutations through the interaction of NaV1.5 α-subunits. *Cardiovasc. Res.* **96**, 53–63 (2012).

26. Holst, A. G. *et al.* Sick sinus syndrome, progressive cardiac conduction disease, atrial flutter and ventricular tachycardia caused by a novel SCN5A mutation. *Cardiol.* **115**, 311–316 (2010).

27. Swan, H. *et al.* A gain-of-function mutation of the SCN5A gene causes exercise-induced polymorphic ventricular arrhythmias. *Circ. Cardiovasc. Genet.* **7**, 771–781 (2014).

28. Gui, J. *et al.* Multiple loss-of-function mechanisms contribute to SCN5A-related familial sick sinus syndrome. *PLOS ONE* **5**, e10985 (2010).

29. Gui, J., Wang, T., Trump, D., Zimmer, T. & Lei, M. Mutation-specific effects of polymorphism H558R in SCN5A-related sick sinus syndrome. *J. Cardiovasc. Electrophysiol.* **21**, 564–573 (2010).

30. Smits, J. P. *et al.* A mutation in the human cardiac sodium channel (E161K) contributes to sick sinus syndrome, conduction disease and Brugada syndrome in two families. *J. Mol. Cell. Cardiol.* **38**, 969–981 (2005).

31. Makita, N. *et al.* Congenital atrial standstill associated with coinheritance of a novel SCN5A mutation and connexin 40 polymorphisms. *Heart Rhythm.* **2**, 1128–1134 (2005).

32. Marangoni, S. *et al.* A Brugada syndrome mutation (p.S216L) and its modulation by p.H558R polymorphism: standard and dynamic characterization. *Cardiovasc. Res.* **91**, 606–616 (2011).

33. Wang, D. W. *et al.* Cardiac sodium channel dysfunction in sudden infant death syndrome. *Circulation* **115**, 368–376 (2007).

34. Abe, K. *et al.* Sodium channelopathy underlying familial sick sinus syndrome with early onset and predominantly male characteristics. *Circ. Arrhythm. Electrophysiol.* **7**, 511–517 (2014).

35. Chen, L., Santarelli, V., Horn, R. & Kallen, R. A unique role for the S4 segment of domain 4 in the inactivation of sodium channels. *J. Gen. Physiol.* **108**, 549–556 (1996).

36. Gosselin-Badaroudine, P. *et al.* A proton leak current through the cardiac sodium channel is linked to mixed arrhythmia and the dilated cardiomyopathy phenotype. *PLOS ONE* **7**, e38331 (2012).

37. Bezzina, C. R. *et al.* Compound heterozygosity for mutations (W156X and R225W) in SCN5A associated with severe cardiac conduction disturbances and degenerative changes in the conduction system. *Circ. Res.* **92**, 159–168 (2003).

38. Watanabe, H. *et al.* Electrocardiographic characteristics and SCN5A mutations in idiopathic ventricular fibrillation associated with early repolarization. *Circ. Arrhythm. Electrophysiol.* **4**, 874–881 (2011).

39. Neu, A. *et al.* A homozygous SCN5A mutation in a severe, recessive type of cardiac conduction disease. *Hum. Mutat.* **31**, E1609–E1621 (2010).

40. Calloe, K. *et al.* Multiple arrhythmic syndromes in a newborn, owing to a novel mutation in SCN5A. *Can. J. Physiol. Pharmacol.* **89**, 723–736 (2011).

41. Itoh, H., Shimizu, M., Mabuchi, H. & Imoto, K. Clinical and electrophysiological characteristics of Brugada syndrome caused by a missense mutation in the S5-pore site of SCN5A. *J. Cardiovasc. Electrophysiol.* **16**, 378–383 (2005).

42. Poelzing, S. *et al.* SCN5A polymorphism restores trafficking of a Brugada syndrome mutation on a separate gene. *Circulation* **114**, 368–376 (2006).

43. Shinlapawittayatorn, K. *et al.* A novel strategy using cardiac sodium channel polymorphic fragments to rescue trafficking-deficient SCN5A mutations. *Circ. Cardiovasc. Genet.* **4**, 500–509 (2011).

44. Saito, Y. A. *et al.* Sodium channel mutation in irritable bowel syndrome: evidence for an ion channelopathy. *Am. J. Physiol. Gastrointest. Liver Physiol.* **296**, G211–G218 (2009).

45. Wang, D. W., Viswanathan, P. C., Balser, J. R., George, A. L. & Benson, D. W. Clinical, genetic, and biophysical characterization of SCN5A mutations associated with atrioventricular conduction block. *Circulation* **105**, 341–346 (2002).

46. Keller, D. I. *et al.* Brugada syndrome and fever: genetic and molecular characterization of patients carrying SCN5A mutations. *Cardiovasc. Res.* **67**, 510–519 (2005).

47. Cordeiro, J. M. *et al.* Compound heterozygous mutations P336L and I1660V in the human cardiac sodium channel associated with the Brugada syndrome. *Circulation* **114**, 2026–2033 (2006).

48. Olesen, M. S. *et al.* High prevalence of long QT syndrome associated SCN5A variants in patients with early-onset lone atrial fibrillation. *Circ. Cardiovasc. Genet.* **5**, 450 (2012).

49. Vatta, M. *et al.* Novel mutations in domain I of SCN5A cause Brugada syndrome. *Mol. Genet. Metab.* **75**, 317–324 (2002).

50. Guo, Q. *et al.* A novel mutation in the SCN5A gene contributes to arrhythmogenic characteristics of early repolarization syndrome. *Int. J. Mol. Med.* **37**, 727–733 (2016).

51. Pfahnl, A. E. *et al.* A sodium channel pore mutation causing Brugada syndrome. *Heart Rhythm.* **4**, 46–53 (2007).

52. Zhang, J. *et al.* Electrophysiological and trafficking defects of the SCN5A T353I mutation in Brugada syndrome are rescued by alpha-allocryptopine. *Eur. J. Pharmacol.* **746**, 333–343 (2015).

53. Hong, K. *et al.* Phenotypic characterization of a large European family with Brugada syndrome displaying a sudden unexpected death syndrome mutation in SCN5A. *J. Cardiovasc. Electrophysiol.* **15**, 64–69 (2004).

54. Vatta, M. *et al.* Genetic and biophysical basis of sudden unexplained nocturnal death syndrome (SUNDS), a disease allelic to Brugada syndrome. *Hum. Mol. Genet.* **11**, 337–345 (2002).

55. Detta, N. *et al.* Genetic analysis in a family affected by sick sinus syndrome may reduce the sudden death risk in a young aspiring competitive athlete. *Int. J. Cardiol.* **170**, e63–e65 (2014).

56. Frustaci, A. *et al.* Cardiac histological substrate in patients with clinical phenotype of Brugada syndrome. *Circulation* **112**, 3680–3687 (2005).

57. Rossenbacker, T. *et al.* Novel pore mutation in SCN5A manifests as a spectrum of phenotypes ranging from atrial flutter, conduction disease, and Brugada syndrome to sudden cardiac death. *Heart Rhythm.* **1**, 610–615 (2004).

58. Hu, D. *et al.* Novel mutation in the SCN5A gene associated with arrhythmic storm development during acute myocardial infarction. *Heart Rhythm.* **4**, 1072–1080 (2007).

59. Kato, K. *et al.* Cardiac channelopathies associated with infantile fatal ventricular arrhythmias: from the cradle to the bench. *J. Cardiovasc. Electrophysiol.* **25**, 66–73 (2014).

60. Itoh, H., Shimizu, M., Takata, S., Mabuchi, H. & Imoto, K. A novel missense mutation in the SCN5A gene associated with Brugada syndrome bidirectionally affecting blocking actions of antiarrhythmic drugs. *J. Cardiovasc. Electrophysiol.* **16**, 486–493 (2005).

61. Itoh, H. *et al.* A paradoxical effect of lidocaine for the N406S mutation of SCN5A associated with Brugada syndrome. *Int. J. Cardiol.* **121**, 239–248 (2007).

62. Horne, A. J., Eldstrom, J., Sanatani, S. & Fedida, D. A novel mechanism for LQT3 with 2:1 block: a pore-lining mutation in NaV1.5 significantly affects voltage-dependence of activation. *Heart Rhythm.* **8**, 770–777 (2011).

63. Crotti, L. *et al.* Torsades de pointes following acute myocardial infarction: evidence for a deadly link with a common genetic variant. *Heart Rhythm.* **9**, 1104–1112 (2012).

64. Holst, A. G. *et al.* A novel SCN5A mutation in a patient with coexistence of Brugada syndrome traits and ischaemic heart disease. *Case Rep. Med.* **2009** (2009).

65. Viswanathan, P. C., Benson, D. W. & Balser, J. R. A common SCN5A polymorphism modulates the biophysical effects of an SCN5A mutation. *J. Clin. Investig.* **111**, 341–346 (2003).

66. Tan, H. L. *et al.* A sodium-channel mutation causes isolated cardiac conduction disease. *Nature* **409**, 1043–1047 (2001).

67. Shuraih, M. *et al.* A common SCN5A variant alters the responsiveness of human sodium channels to class I antiarrhythmic agents. *J. Cardiovasc. Electrophysiol.* **18**, 434–440 (2007).

68. Aiba, T. *et al.* A mutation causing Brugada syndrome identifies a mechanism for altered autonomic and oxidant regulation of cardiac sodium currents. *Circ. Cardiovasc. Genet.* **7**, 249–256 (2014).

69. Otagiri, T. *et al.* Cardiac ion channel gene mutations in sudden infant death syndrome. *Pediatr. Res.* **64**, 482–487 (2008).

70. Chiang, K.-C., Lai, L.-P. & Shieh, R.-C. Characterization of a novel NaV1.5 channel mutation, A551T, associated with Brugada syndrome. *J. Biomed. Sci.* **16**, 1 (2009).

71. Juang, J.-M. J. *et al.* Utilizing multiple in silico analyses to identify putative causal SCN5A variants in Brugada syndrome. *Sci. Rep.* **4**, 3850 (2014).

72. Kauferstein, S., Kiehne, N., Peigneur, S., Tytgat, J. & Bratzke, H. Cardiac channelopathy causing sudden death as revealed by molecular autopsy. *Int. J. Leg. Med.* **127**, 145–151 (2013).

73. Murphy, L. L. *et al.* Developmentally regulated SCN5A splice variant potentiates dysfunction of a novel mutation associated with severe fetal arrhythmia. *Heart Rhythm.* **9**, 590–597 (2012).

74. Surber, R. *et al.* Combination of cardiac conduction disease and long QT syndrome caused by mutation T1620K in the cardiac sodium channel. *Cardiovasc. Res.* **77**, 740–748 (2008).

75. Wan, X., Chen, S., Sadeghpour, A., Wang, Q. & Kirsch, G. E. Accelerated inactivation in a mutant Na+ channel associated with idiopathic ventricular fibrillation. *Am. J. Physiol. Heart Circ. Physiol.* **280**, H354–H360 (2001).

76. Glynn, P. *et al.* Voltage-gated sodium channel phosphorylation at ser571 regulates late current, arrhythmia, and cardiac function in vivo. *Circulation* **132**, 567–577 (2015).

77. Albert, C. M. *et al.* Cardiac sodium channel gene variants and sudden cardiac death in women. *Circulation* **117**, 16–23 (2008).

78. Tester, D. J. *et al.* Epidemiologic, molecular, and functional evidence suggest A572D-SCN5A should not be considered an independent LQT3-susceptibility mutation. *Heart Rhythm.* **7**, 912–919 (2010).

79. Yang, P. *et al.* Allelic variants in long-QT disease genes in patients with drug-associated torsades de pointes. *Circulation* **105**, 1943–1948 (2002).

80. Wehrens, X. H. *et al.* A novel mutation L619F in the cardiac Na+ channel SCN5A associated with long-QT syndrome (LQT3): a role for the I-II linker in inactivation gating. *Hum. Mutat.* **21**, 552–552 (2003).

81. Calloe, K. *et al.* Characterization and mechanisms of action of novel NaV1.5 channel mutations associated with Brugada syndrome. *Circ. Arrhythm. Electrophysiol.* **6**, 177–184 (2013).

82. Cheng, J. *et al.* The common African American polymorphism SCN5A-S1103Y interacts with mutation SCN5A-R680H to increase late Na current. *Physiol. Genomics* **43**, 461–466 (2011).

83. Mok, N.-S. *et al.* A newly characterized SCN5A mutation underlying Brugada syndrome unmasked by hyperthermia. *J. Cardiovasc. Electrophysiol.* **14**, 407–411 (2003).

84. Sottas, V. *et al.* Characterization of 2 genetic variants of NaV1.5-Arginine 689 found in patients with cardiac arrhythmias. *J. Cardiovasc. Electrophysiol.* **24**, 1037–1046 (2013).

85. Hong, K., Hu, J., Yu, J. & Brugada, R. Concomitant Brugada-like and short QT electrocardiogram linked to SCN5A mutation. *Eur. J. Hum. Genet.* **20**, 1189–1192 (2012).

86. Potet, F. *et al.* Novel Brugada SCN5A mutation leading to ST segment elevation in the inferior or the right precordial leads. *J. Cardiovasc. Electrophysiol.* **14**, 200–203 (2003).

87. Wang, L. *et al.* De novo mutation in the SCN5A gene associated with Brugada syndrome. *Cell. Physiol. Biochem.* **36**, 2250–2262 (2015).

88. Nguyen, T. P., Wang, D. W., Rhodes, T. H. & George, A. L. Divergent biophysical defects caused by mutant sodium channels in dilated cardiomyopathy with arrhythmia. *Circ. Res.* **102**, 364–371 (2008).

89. Kinoshita, K. *et al.* SCN5A (K817E), a novel Brugada syndrome–associated mutation that alters the activation gating of NaV1.5 channel. *Heart Rhythm.* **13**, 1113–1120 (2016).

90. David, M. *et al.* Augmented window INa by the novel SCN5A mutation L828F: Implications for abnormal ventricular impulse formation and treatment. In *Heart Rhythm Society*, AB04–02 (2012).

91. Zhang, Y. *et al.* Correlations between clinical and physiological consequences of the novel mutation R878C in a highly conserved pore residue in the cardiac Na+ channel. *Acta Physiol.* **194**, 311–323 (2008).

92. Tarradas, A. *et al.* A novel missense mutation, I890T, in the pore region of cardiac sodium channel causes Brugada syndrome. *PLOS ONE* **8**, e53220 (2013).

93. Wang, S.-Y., Tikhonov, D. B., Mitchell, J., Zhorov, B. & Wang, G. K. Irreversible block of cardiac mutant Na+ channels by batrachotoxin. *Channels* **1**, 179–188 (2007).

94. Ruan, Y., Liu, N., Bloise, R., Napolitano, C. & Priori, S. G. Gating properties of SCN5A mutations and the response to mexiletine in long-QT syndrome type 3 patients. *Circulation* **116**, 1137–1144 (2007).

95. Schwartz, P. J. *et al.* A molecular link between the sudden infant death syndrome and the long-QT syndrome. *New Engl. J. Med.* **343**, 262–267 (2000).

96. Hsueh, C.-H. *et al.* Distinct functional defect of three novel Brugada syndrome related cardiac sodium channel mutations. *J. Biomed. Sci.* **16**, 1 (2009).

97. Hayashi, K. *et al.* Functional characterization of rare variants implicated in susceptibility to lone atrial fibrillation. *Circ. Arrhythm. Electrophysiol.* **8**, 1095–1104 (2015).

98. Hu, D. *et al.* Dual variation in SCN5A and CACNB2b underlies the development of cardiac conduction disease without Brugada syndrome. *Pacing Clin. Electrophysiol.* **33**, 274–285 (2010).

99. Mohler, P. J. *et al.* NaV1.5 E1053K mutation causing Brugada syndrome blocks binding to ankyrin-G and expression of NaV1.5 on the surface of cardiomyocytes. *Proc. Natl. Acad. Sci. U.S.A.* **101**, 17533–17538 (2004).

100. Splawski, I. *et al.* Variant of SCN5A sodium channel implicated in risk of cardiac arrhythmia. *Science* **297**, 1333–1336 (2002).

101. Winkel, B. G. *et al.* The prevalence of mutations in KCNQ1, KCNH2, and SCN5A in an unselected national cohort of young sudden unexplained death cases. *J. Cardiovasc. Electrophysiol.* **23**, 1092–1098 (2012).

102. Ge, J. *et al.* Molecular and clinical characterization of a novel SCN5A mutation associated with atrioventricular block and dilated cardiomyopathy. *Circ. Arrhythm. Electrophysiol.* **1**, 83–92 (2008).

103. Huang, H., Zhao, J., Barrane, F.-Z., Champagne, J. & Chahine, M. NaV1.5/R1193Q polymorphism is associated with both long QT and Brugada syndromes. *Can. J. Cardiol.* **22**, 309–313 (2006).

104. Wang, Q. *et al.* The common SCN5A mutation R1193Q causes LQTS-type electrophysiological alterations of the cardiac sodium channel. *J. Med. Genet.* **41**, e66–e66 (2004).

105. Medeiros-Domingo, A. *et al.* Unique mixed phenotype and unexpected functional effect revealed by novel compound heterozygosity mutations involving SCN5A. *Heart Rhythm.* **6**, 1170–1175 (2009).

106. Groenewegen, W. A. *et al.* A cardiac sodium channel mutation cosegregates with a rare connexin 40 genotype in familial atrial standstill. *Circ. Res.* **92**, 14–22 (2003).

107. Watanabe, H. *et al.* Striking in vivo phenotype of a disease-associated human SCN5A mutation producing minimal changes in vitro. *Circulation* **124**, 1001–1011 (2011).

108. Abriel, H. *et al.* Novel arrhythmogenic mechanism revealed by a long-QT syndrome mutation in the cardiac Na+ channel. *Circ. Res.* **88**, 740–745 (2001).

109. Wang, H.-G. *et al.* A novel NaV1.5 voltage sensor mutation associated with severe atrial and ventricular arrhythmias. *J. Mol. Cell. Cardiol.* **92**, 52–62 (2016).

110. Casini, S. *et al.* Characterization of a novel SCN5A mutation associated with Brugada syndrome reveals involvement of DIIIS4–S5 linker in slow inactivation. *Cardiovasc. Res.* **76**, 418–429 (2007).

111. Wang, D. W., Yazawa, K., George, A. L. & Bennett, P. B. Characterization of human cardiac Na+ channel mutations in the congenital long QT syndrome. *Proc. Natl. Acad. Sci. U.S.A.* **93**, 13200–13205 (1996).

112. Yong, S. L. *et al.* Characterization of the cardiac sodium channel SCN5A mutation, N1325S, in single murine ventricular myocytes. *Biochem. Biophys. Res. Commun.* **352**, 378–383 (2007).

113. Wedekind, H. *et al.* De novo mutation in the SCN5A gene associated with early onset of sudden infant death. *Circulation* **104**, 1158–1164 (2001).

114. Smits, J. P. *et al.* Substitution of a conserved alanine in the domain IIIS4–S5 linker of the cardiac sodium channel causes long QT syndrome. *Cardiovasc. Res.* **67**, 459–466 (2005).

115. Huang, H. *et al.* Biophysical characterization of a new SCN5A mutation S1333Y in a SIDS infant linked to long QT syndrome. *FEBS Lett.* **583**, 890–896 (2009).

116. Samani, K. *et al.* A novel SCN5A mutation V1340I in Brugada syndrome augmenting arrhythmias during febrile illness. *Heart Rhythm.* **6**, 1318–1326 (2009).

117. Keller, D. I. *et al.* A novel SCN5A mutation, F1344S, identified in a patient with Brugada syndrome and fever-induced ventricular fibrillation. *Cardiovasc. Res.* **70**, 521–529 (2006).

118. Kyndt, F. *et al.* Novel SCN5A mutation leading either to isolated cardiac conduction defect or Brugada syndrome in a large French family. *Circulation* **104**, 3081–3086 (2001).

119. Tan, B.-H., Valdivia, C. R., Song, C. & Makielski, J. C. Partial expression defect for the SCN5A missense mutation G1406R depends on splice variant background Q1077 and rescue by mexiletine. *Am. J. Physiol. Heart Circ. Physiol.* **291**, H1822–H1828 (2006).

120. Baroudi, G. *et al.* Novel mechanism for Brugada syndrome defective surface localization of an SCN5A mutant (R1432G). *Circ. Res.* **88**, e78–e83 (2001).

121. Deschênes, I. *et al.* Electrophysiological characterization of SCN5A mutations causing long QT (E1784K) and Brugada (R1512W and R1432G) syndromes. *Cardiovasc. Res.* **46**, 55–65 (2000).

122. Six, I. *et al.* The occurrence of Brugada syndrome and isolated cardiac conductive disease in the same family could be due to a single SCN5A mutation or to the accidental association of both diseases. *Europace* **10**, 79–85 (2008).

123. Sarhan, M. F., Van Petegem, F. & Ahern, C. A. A double tyrosine motif in the cardiac sodium channel domain III-IV linker couples calcium-dependent calmodulin binding to inactivation gating. *J. Biol. Chem.* **284**, 33265–33274 (2009).

124. Bankston, J. R. *et al.* A novel and lethal de novo LQT-3 mutation in a newborn with distinct molecular pharmacology and therapeutic response. *PLOS ONE* **2**, e1258 (2007).

125. Ruan, Y. *et al.* Trafficking defects and gating abnormalities of a novel SCN5A mutation question gene-specific therapy in long QT syndrome type 3. *Circ. Res.* **106**, 1374–1383 (2010).

126. Moreau, A. *et al.* Sodium overload due to a persistent current that attenuates the arrhythmogenic potential of a novel LQT3 mutation. *Front. Pharmacol.* **4**, 126 (2013).

127. Li, Q. *et al.* Gain-of-function mutation of NaV1.5 in atrial fibrillation enhances cellular excitability and lowers the threshold for action potential firing. *Biochem. Biophys. Res. Commun.* **380**, 132–137 (2009).

128. Saber, S. *et al.* Complex genetic background in a large family with Brugada syndrome. *Physiol. Rep.* **3**, e12256 (2015).

129. Rook, M. B. *et al.* Human SCN5A gene mutations alter cardiac sodium channel kinetics and are associated with the Brugada syndrome. *Cardiovasc. Res.* **44**, 507–517 (1999).

130. Baroudi, G., Carbonneau, E., Pouliot, V. & Chahine, M. SCN5A mutation (T1620M) causing Brugada syndrome exhibits different phenotypes when expressed in Xenopus oocytes and mammalian cells. *FEBS Lett.* **467**, 12–16 (2000).

131. Shirai, N. *et al.* A mutant cardiac sodium channel with multiple biophysical defects associated with overlapping clinical features of Brugada syndrome and cardiac conduction disease. *Cardiovasc. Res.* **53**, 348–354 (2002).

132. Wang, D. W., Makita, N., Kitabatake, A., Balser, J. R. & George, A. L. Enhanced Na+ channel intermediate inactivation in Brugada syndrome. *Circ. Res.* **87**, e37–e43 (2000).

133. Kambouris, N. G. *et al.* A revised view of cardiac sodium channel "blockade" in the long-QT syndrome. *J. Clin. Investig.* **105**, 1133–1140 (2000).

134. Makita, N. *et al.* A de novo missense mutation of human cardiac Na+ channel exhibiting novel molecular mechanisms of long QT syndrome. *FEBS Lett.* **423**, 5–9 (1998).

135. Tsurugi, T. *et al.* Differential modulation of late sodium current by protein kinase A in R1623Q mutant of LQT3. *Life Sci.* **84**, 380–387 (2009).

136. Zeng, Z. *et al.* Electrophysiological characteristics of a SCN5A voltage sensors mutation R1629Q associated with Brugada syndrome. *PLOS ONE* **8**, e78382 (2013).

137. Wang, D. W. *et al.* Malignant perinatal variant of long-QT syndrome caused by a profoundly dysfunctional cardiac sodium channel. *Circ. Arrhythm. Electrophysiol.* **1**, 370–378 (2008).

138. Nakajima, T. *et al.* Enhanced fast-inactivated state stability of cardiac sodium channels by a novel voltage sensor SCN5A mutation, R1632C, as a cause of atypical Brugada syndrome. *Heart Rhythm.* **12**, 2296–2304 (2015).

139. Núñez, L. *et al.* p.D1690N NaV1.5 rescues p.G1748D mutation gating defects in a compound heterozygous Brugada syndrome patient. *Heart Rhythm.* **10**, 264–272 (2013).

140. Akai, J. *et al.* A novel SCN5A mutation associated with idiopathic ventricular fibrillation without typical ECG findings of Brugada syndrome. *FEBS Lett.* **479**, 29–34 (2000).

141. Amin, A., Verkerk, A., Bhuiyan, Z., Wilde, A. & Tan, H. Novel Brugada syndrome-causing mutation in ion-conducting pore of cardiac Na+ channel does not affect ion selectivity properties. *Acta Physiol. Scand.* **185**, 291–301 (2005).

142. Baroudi, G., Napolitano, C., Priori, S. G., Del Bufalo, A. & Chahine, M. Loss of function associated with novel mutations of the SCN5A gene in patients with Brugada syndrome. *Can. J. Cardiol.* **20**, 425–430 (2004).

143. Valdivia, C. R. *et al.* A trafficking defective, Brugada syndrome-causing SCN5A mutation rescued by drugs. *Cardiovasc. Res.* **62**, 53–62 (2004).

144. Chang, C.-C. *et al.* A novel SCN5A mutation manifests as a malignant form of long QT syndrome with perinatal onset of tachycardia/bradycardia. *Cardiovasc. Res.* **64**, 268–278 (2004).

145. Valdivia, C. R. *et al.* A novel SCN5A arrhythmia mutation, M1766L, with expression defect rescued by mexiletine. *Cardiovasc. Res.* **55**, 279–289 (2002).

146. Groenewegen, W. A. *et al.* A novel LQT3 mutation implicates the human cardiac sodium channel domain IVS6 in inactivation kinetics. *Cardiovasc. Res.* **57**, 1072–1078 (2003).

147. Rivolta, I. *et al.* A novel SCN5A mutation associated with long QT-3: altered inactivation kinetics and channel dysfunction. *Physiol. Genomics* **10**, 191–197 (2002).

148. Lupoglazoff, J. *et al.* Homozygous SCN5A mutation in long-QT syndrome with functional two-to-one atrioventricular block. *Circ. Res.* **89**, e16–e21 (2001).

149. Hu, D. *et al.* ABCC9 is a novel Brugada and early repolarization syndrome susceptibility gene. *Int. J. Cardiol.* **171**, 431–442 (2014).

150. Makita, N. *et al.* The E1784K mutation in SCN5A is associated with mixed clinical phenotype of type 3 long QT syndrome. *J. Clin. Investig.* **118**, 2219–2229 (2008).

151. Wei, J. *et al.* Congenital long-QT syndrome caused by a novel mutation in a conserved acidic domain of the cardiac Na+ channel. *Circulation* **99**, 3165–3171 (1999).

152. Hu, R.-M. *et al.* Arrhythmogenic biophysical phenotype for SCN5A mutation S1787N depends upon splice variant background and intracellular acidosis. *PLOS ONE* **10**, e0124921 (2015).

153. Rivolta, I. *et al.* Inherited Brugada and long QT-3 syndrome mutations of a single residue of the cardiac sodium channel confer distinct channel and clinical phenotypes. *J. Biol. Chem.* **276**, 30623–30630 (2001).

154. Tateyama, M., Kurokawa, J., Terrenoire, C., Rivolta, I. & Kass, R. Stimulation of protein kinase C inhibits bursting in disease-linked mutant human cardiac sodium channels. *Circulation* **107**, 3216–3222 (2003).

155. Liu, K., Yang, T., Viswanathan, P. C. & Roden, D. M. New mechanism contributing to drug-induced arrhythmia rescue of a misprocessed LQT3 mutant. *Circulation* **112**, 3239–3246 (2005).

156. Makita, N. *et al.* Drug-induced long-QT syndrome associated with a subclinical SCN5A mutation. *Circulation* **106**, 1269–1274 (2002).

157. Musa, H. *et al.* SCN5A variant that blocks fibroblast growth factor homologous factor regulation causes human arrhythmia. *Proc. Natl. Acad. Sci. U.S.A.* **112**, 12528–12533 (2015).

158. Petitprez, S. *et al.* Analyses of a novel SCN5A mutation (C1850S): conduction vs. repolarization disorder hypotheses in the Brugada syndrome. *Cardiovasc. Res.* **78**, 494–504 (2008).

159. Makiyama, T. *et al.* A novel SCN5A gain-of-function mutation M1875T associated with familial atrial fibrillation. *J. Am. Coll. Cardiol.* **52**, 1326–1334 (2008).

160. Bankston, J. R. *et al.* A novel LQT-3 mutation disrupts an inactivation gate complex with distinct rate-dependent phenotypic consequences. *Channels* **1**, 273–280 (2007).

161. Glaaser, I. W. *et al.* Perturbation of sodium channel structure by an inherited long QT syndrome mutation. *Nat. Commun.* **3**, 706 (2012).

162. Tan, H. L. *et al.* A calcium sensor in the sodium channel modulates cardiac excitability. *Nature* **415**, 442–447 (2002).

163. Shinlapawittayatorn, K. *et al.* A common SCN5A polymorphism modulates the biophysical defects of SCN5A mutations. *Heart Rhythm.* **8**, 455–462 (2011).

164. Ellinor, P. T. *et al.* Cardiac sodium channel mutation in atrial fibrillation. *Heart Rhythm.* **5**, 99–105 (2008).

165. Bébarová, M. *et al.* Subepicardial phase 0 block and discontinuous transmural conduction underlie right precordial ST-segment elevation by a SCN5A loss-of-function mutation. *Am. J. Physiol. Heart Circ. Physiol.* **295**, H48–H58 (2008).

166. Chen, J. *et al.* Cardiac sodium channel mutation associated with epinephrine-induced QT prolongation and sinus node dysfunction. *Heart Rhythm.* **13**, 289–298 (2016).

167. Shy, D. *et al.* PDZ domain–binding motif regulates cardiomyocyte compartment-specific NaV1.5 channel expression and function. *Circulation* **130**, 147–160 (2014).
